# Human-Constrained AI-Assisted Identification of Defect-Topology Relations for Mechanical Degradation in Porous Solids: A Discrete-Element Study

**DOI:** 10.3390/ma19173756

**Published:** 2026-09-03

**Authors:** Yi Zhang, Yifei Liu, Heping Xie

**Affiliations:** Institute of Deep Earth Sciences and Green Energy, College of Civil and Transportation Engineering, Shenzhen University, Shenzhen 518060, China; 2023095051@email.szu.edu.cn (Y.Z.); xiehp@szu.edu.cn (H.X.)

**Keywords:** porous brittle solids, defect topology, strength decay, modulus decay, interpretable relation, discrete-element method

## Abstract

Porosity measures how much material is removed but not how defects interrupt load-bearing paths. We develop a human-constrained, AI-assisted workflow to identify compact topology–degradation relations in a controlled bonded-particle system with 10,040 specimens spanning five pure families and mixtures. Investigators defined the descriptor grammar, admissible information, physical prior, model size, and approval rules; language models proposed and critiqued descriptor concepts; authors curated and implemented the 127-channel bench; deterministic TRAIN-only code selected terms and fitted the ordinary-least-squares relations. On the reused, non-independent reporting benchmark, the six-descriptor representations produced higher scores than the evaluated three-dimensional convolutional network under the matched protocol. Applied without reselection or refitting to 900 porous specimens from three post-freeze packing realizations, the relations achieved prospective numerical $R^{2}=0.516$ for strength-decay rate and 0.735 for modulus-decay rate, with greater family heterogeneity for strength. Across families, modulus-decay rate was predicted more consistently than strength-decay rate. Together with the exact contrast $k=k_{E}+\Delta k$, this predictive asymmetry motivates, but does not establish, a two-stage failure hypothesis. The exponential form remains a compact empirical parameterization rather than an established physical law. All evidence is numerical and confined to the tested YADE/JCFpm setting; validation across numerical configurations and experimental systems remains necessary.

## 1. Introduction

Defect porosity (hereafter porosity) is a primary control on the mechanical response of brittle porous media. Classical relations express compressive strength and elastic modulus as exponential or power-law functions of porosity [1,2,3,4,5,6]. Their utility lies in collapsing a dominant volume effect into one scalar. That scalar is insufficient whenever equal-porosity structures differ in connectivity, orientation, clustering or load-bearing ligament geometry. A finite-element study, for example, found different elastic responses for regular and irregular pore arrangements [7]. Separately, compression tests on recycled-aggregate concrete with preformed pores, combined with X-ray computed tomography, related porosity and pore size to strength, modulus and crack development [8]. The unresolved problem is therefore not whether geometry beyond porosity matters, but how to represent its mechanically relevant three-dimensional content compactly and auditably.

Integral geometry, correlation functions, graph summaries and topological data analysis already provide routes beyond porosity. Minkowski functionals have been linked to strength degradation; multipoint correlations distinguish mechanically consequential morphology; local Morse–Smale constructions extract pore and ligament graphs; and physics-based bottleneck, channel and dominant-path descriptors improve structure–property prediction [9,10,11,12]. Recent direction-aware topological descriptors further show that embedding the loading axis can approach voxel-model accuracy for Young’s modulus [13]. Graph-theoretical texture descriptors and evolving force paths have likewise been related to brittle-porous response, damage localization and fracture-path prediction [14,15,16]. Scalar Minkowski measures do not retain direction-resolved bottlenecks or multiscale spatial organization, whereas graph, correlation and topological methods require an explicit choice of representation and summary. The novelty pursued here is the registered synthesis and audit of those information classes, followed by a paired strength–modulus test of a compact representation; no novelty is claimed for any individual morphology statistic, bottleneck concept or directional descriptor.

Three-dimensional convolutional networks and morphology-informed neural models can learn directly from voxelized heterogeneous structures [17,18]. They trade explicit representation design for end-to-end feature learning, whereas symbolic regression searches compact expressions in a supplied variable space [19,20,21]. Large language models add a different capability: they can propose candidate mathematical constructions and mechanical rationales, as learned proposal systems have broadened human search in other scientific domains [22,23]. That capability does not make the resulting analysis autonomous. Here, investigators defined the grammar, information boundary, mandatory minimum-cut prior, admissibility rules and model size. Language models generated and criticized descriptor candidates within those boundaries; human review admitted implementations; deterministic scripts alone selected terms and fitted coefficients.

The contribution is the audited combination of three elements. First, a human-defined compositional grammar and language-model-assisted proposal stage expand a physically bounded descriptor space without allowing the model to fit the reported equations. Second, compact topology representations, conventional morphology and raw voxels are compared on the same controlled numerical frame. Third, strength and modulus are analyzed specimen by specimen to test whether static initial topology is equally sufficient for elastic attenuation and peak failure. The study therefore asks two limited questions: whether the compact representation is competitive within the registered DEM setting, and where its explanatory boundary differs between modulus decay rate $k_{E}$ and strength decay rate *k*. It does not test universal material constants or autonomous AI discovery.

## 2. Materials and Methods

### 2.1. Controlled Digital Specimen Library and Mechanical Targets

Specimens in the original corpus were generated and loaded in YADE using the Jointed Cohesive Frictional Particle Model (JCFpm) [24,25]. The author-confirmed local executable was the WSL yadedaily build; its exact historical build identifier was not retained, so the official YADE website is provided instead of substituting the current installation (https://yade-dem.org/, accessed on 29 August 2026). JCFpm is a bonded-particle formulation: initially interacting near neighbors transmit normal and tangential forces through cohesive contacts, bonds fail under the model’s tensile or shear criterion, and subsequent contact is frictional. Progressive bond loss and localization can therefore emerge during compression, but the contact thresholds are not specimen-scale strengths.

The original frozen intact packing contained 14,800 spheres in a 10×10×20 mm domain, with the *z* direction as loading axis. The radius was prescribed as *R*∼U(0.20,0.30) mm, for which $\bar{R}=0.25$ mm and $\mathrm{CV}\left(R\right)=\left(0.30-0.20\right)/\left(\sqrt{12}\;\bar{R}\right)=0.115$. The corresponding mean diameter of 0.50 mm gives approximately 20 mean particle diameters across the 10 mm width and 40 across the 20 mm height. The 0.10 mm span introduces modest polydispersity to suppress the artificial ordering of equal spheres without creating a separate particle-size population, while the mean size retains sufficient contact-network resolution at a tractable cost for the 10,040-case sweep. Thus, 0.20–0.30 mm is a numerical resolution–cost choice rather than a material-specific particle-size calibration; no particle-size convergence claim is made.

Particle density was 2500 kg m^−3^, particle-scale Young’s modulus was 130 GPa, the tangential-to-normal stiffness parameter was 0.3, and the interaction-radius multiplier was 1.1. The $35^{{}^{\circ}}$ interparticle friction angle sets the Coulomb limit for non-cohesive or failed contacts, with $\mu =\tan(35^{{}^{\circ}})$; it is not a measured macroscopic friction angle. Likewise, 50 MPa is the contact tensile-failure threshold and 250 MPa is the contact shear-cohesion parameter, not the uniaxial tensile or compressive strength of a specimen. During the initial numerical calibration stage (Phase 0), they were adjusted together at a fixed 1:5 ratio to place the pore-free uniaxial compressive strength within the numerical acceptance range 60–150 MPa; the retained packing gave 84.35 MPa. This was a numerical compressive calibration, not a macroscopic tensile-strength calibration. Uniaxial compression used a nominal axial strain rate of −0.5 s^−1^, damping 0.4, and a global-stiffness time-step safety factor of 0.5.

Peak compressive stress defined the uniaxial compressive strength $\sigma_{c}$. The pre-peak tangent modulus *E* was the ordinary-least-squares slope over samples between 30% and 50% of $\sigma_{c}$, with a secant fallback when fewer than five samples lay in that interval. The archived intact reference was processed identically and gave $\sigma_{c0}=84.35$ MPa and $E_{0}=38.66$ GPa. Fracture toughness was not an independently prescribed or calibrated macroscopic input. Bond-scale tensile and shear thresholds generate progressive breakage but do not by themselves establish a specimen-scale $K_{\mathrm{IC}}$.

Defect fields were generated on a 40×40×80 Boolean grid with a 0.25 mm voxel edge, and particles whose centres fell in defect voxels were removed. The registered defect-field porosity ϕ is the deletion-region voxel fraction of the nominal specimen. It is distinct from the geometric interparticle void fraction $\phi_{\mathrm{ip}}$ of the intact particle discretization and is the only porosity used in the attenuation transform. Native interparticle voids may contain sharp throats or tips that concentrate local stress, but because the original specimens share one intact packing, their independent contribution was neither varied nor identified. In particular, ϕ and $\phi_{\mathrm{ip}}$ are not added and relabeled as one measured material porosity.

Twenty registered target levels spanned 0.020–0.200. Mechanical transformations used stored realized ϕ, whereas the level-wise summaries reported below use the registered target levels only to align observations. Boolean and Clustered fields are pore-like; Chain paths used case-seeded isotropic initial directions, 0.4 mm steps and persistence 0.98; and network fields followed a random geometric graph. Pure DFN fields used circular disks of radius 1.8 mm and finite numerical thickness 0.5 mm (two voxels), with isotropic disk normals. Mixed specimens were unions of all five generators; their case-level DFN preferred axes were sampled isotropically, while orientation concentrations and component weights were stored in the manifest. Thus, orientation relative to the *z* loading axis enters through the generated geometry and registered anisotropy descriptors, not through response-dependent adjustment. The finite-thickness DFN fields represent crack-like deletion zones, not zero-aperture discontinuities or frictional crack surfaces, and the deletion construction does not resolve a specimen-scale fracture-process zone.

Five pure families represented Boolean pores, chains, connected networks, disk-fracture networks (DFN), and clustered defects; Mixed combined all five. Family, target porosity ($0.02\leq \phi_{\mathrm{target}}\leq 0.20$, maximum realized 0.2079) and seed define a reproducible case. Table 1 records the three ledgers and shows that both extensions added TRAIN cases only, leaving VAL and TEST membership unchanged.

**Figure 1:**
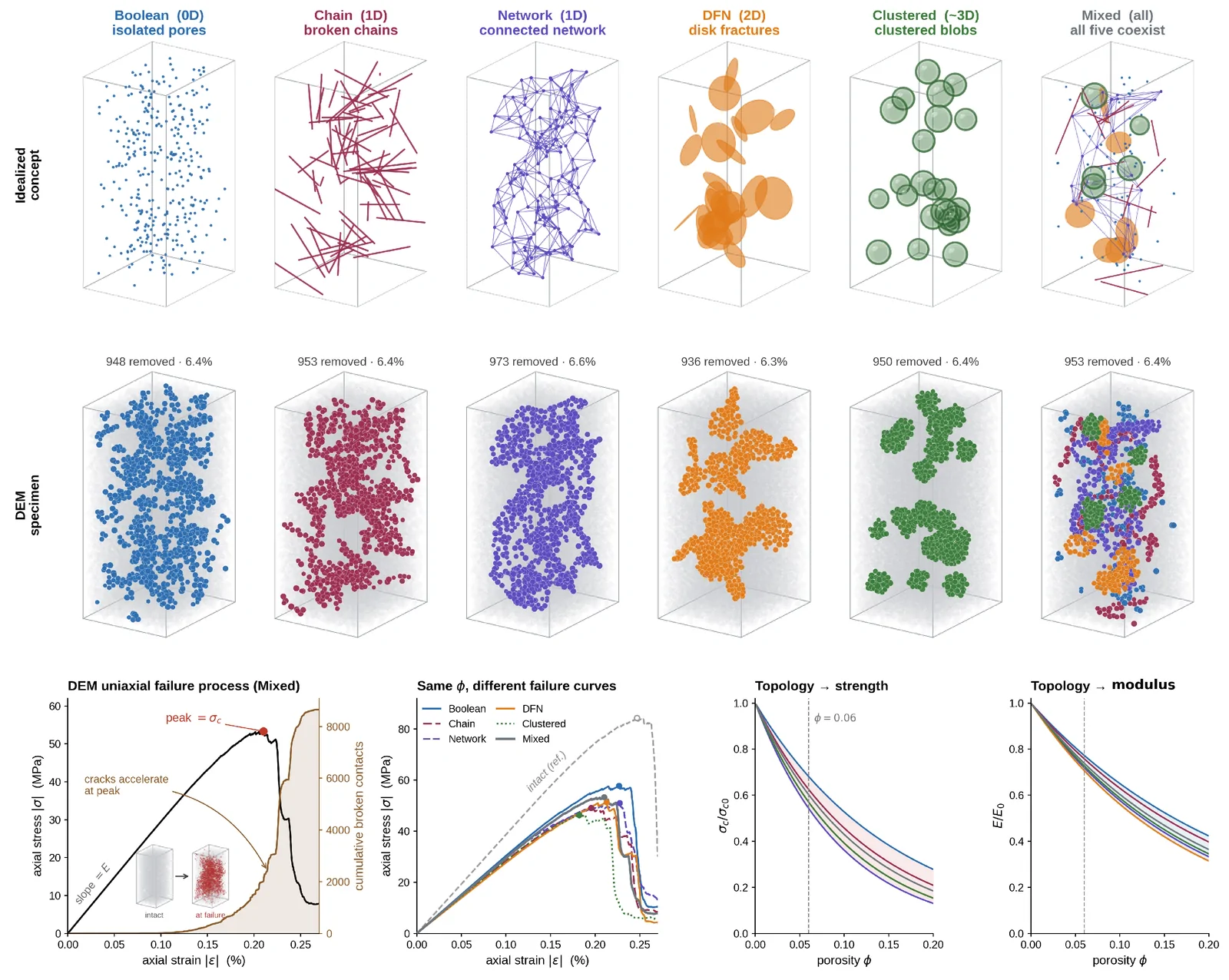
Controlled digital specimens isolate defect topology. (**Top**) Idealized defect fields. Boolean and Clustered fields are pore-like; DFN disks are 0.5 mm (two-voxel) finite-thickness crack-like deletion zones, not zero-aperture discontinuities or frictional crack surfaces. Mixed combines all five generators. (**Middle**) Particle-resolved specimens near ϕ=0.06; registered ϕ is deletion-field porosity and excludes the intact packing’s interparticle void. (**Bottom**) Extraction of peak strength $\sigma_{c}$ and pre-peak modulus *E*, bond-break accumulation, and illustrative responses. Across the bottom row, line colors identify the six defect families; filled circles mark peak stresses, small red dots in the failure inset mark broken contacts, the gray dashed curve is the intact reference, and vertical gray dashed lines mark ϕ=0.06. Family, target level and seed determine each removed-particle field. Curves are normalized at ϕ=0; simulated defect levels begin at 0.02. Renderings and responses are illustrative, not matched quantitative comparisons.

Figure 1 summarizes the controlled defect families and representative mechanical responses.

The retained strength and modulus were written in exponential form,

(1) $$\frac{\sigma_{c}}{\sigma_{c0}}=\exp\left(-\phi k\right),\;\frac{E}{E_{0}}=\exp\left(-\phi k_{E}\right),$$

which defines the specimen-level decay rates

(2) $$k=-\frac{\ln(\sigma_{c}/\sigma_{c0})}{\phi },\;k_{E}=-\frac{\ln(E/E_{0})}{\phi }.$$

The primary targets were *k* and $k_{E}$, not the porosity-dominated products ϕk and $\phi k_{E}$. For an observed retention r(ϕ), −lnr(ϕ)/ϕ is a secant normalization of log retention from zero to the observed porosity, not evidence of a constant physical derivative. Equation (1) is therefore an exact specimen-level reparameterization once the rate is defined; the empirical assumption enters when a fixed topology representation predicts that rate. We call the targets porosity-normalized, not porosity-independent. A TRAIN-only sensitivity compared the exponential form with ${\left(1-\phi \right)}^{a(g)}$ and $\exp[-\phi^{p}a\left(g\right)]$, selecting p∈[0.5,1.5] only inside nested family–seed-grouped folds and evaluating unclipped original retention. It was repeated within each family with descriptor identities fixed, coefficients refitted and reporting VAL and TEST excluded. Because ${\left(1-\phi \right)}^{a}=\exp\left[-a\left\{\phi +\phi^{2}/2+O\left(\phi^{3}\right)\right\}\right]$, similar forms over ϕ≤0.20 are expected and do not identify a unique mechanism. Shared 1/ϕ scaling was audited by replacing or removing the normalized minimum-cut term.

### 2.2. Human-Defined Grammar and AI-Assisted Descriptor Proposal

To avoid compressing complex defect geometry directly into opaque latent variables, candidate descriptors were constructed through the compositional grammar

(3) f=S∘𝒪∘R(G),

where G is the initial three-dimensional defect geometry; R maps it to binary fields, interfaces, skeletons, or particle–bond graphs; 𝒪 applies operations such as multiscale correlation, structural tensors, connectivity analysis, or path-bottleneck extraction; and S returns means, extrema, integrals, characteristic scales, or normalized statistics. Human investigators defined this grammar, restricted it to initial geometry and loading-direction information, and specified the admissible physical boundaries. Within these constraints, language models assisted candidate ideation and physical critique. The authors then curated, implemented and precomputed the 127 channels admitted to the exploratory bench; the historical record does not retain a channel-level mapping from model output to each registered implementation. Deterministic code evaluated the implemented quantities, while human review controlled whether a candidate could enter the registered pool.

The resulting descriptor language was organized along two complementary axes rather than treated as an unstructured feature list. Spatial support distinguishes pore/ligament-scale, local/multiscale-field, and specimen/network-scale representations; physical information distinguishes size and surface, distribution and heterogeneity, spatial correlation, orientation and anisotropy, connectivity and topology, and load-path bottlenecks. Human protocol lock reduced the broader exploratory language to the pinned minimum-cut term plus 30 optional geometry candidates, yielding the 31-column protocol-locked pool used for deterministic selection. Two-point and integral-geometric statistics follow the broader heterogeneous-materials framework [26]. The full protocol-locked pool is enumerated in the Supplementary Information.

All inputs in the fitted relations are deterministically reconstructible from the defect field and frozen initial particle geometry without stress–strain outcomes. The two relations contain ten unique descriptors: $\left\{k_{\mathrm{mincut}},\rho_{\mathrm{br}}\right\}$ form the shared motif, while four additional descriptors are response specific to each target. Table 2 groups these retained quantities by physical role and gives their compact definitions, construction logic and relation membership. Exact implementation details, numerical conventions and archive-field mappings accompany the Supplementary Information because the fitted coefficients in Table 3 apply only to this registered representation. The public symbol $S_{\mathrm{nem}}$ avoids collision with the conventional two-point probability function $S_{2}\left(r\right)$, and ${\bar{Z}}_{\mathrm{bnd}}$ denotes the reconstructed field verified to be exactly equal to the archived coord_num column.

### 2.3. AI-Assisted Exploration and Human Protocol Lock

On the original 5000-case bench, manual enumeration, language-model-assisted ideation and a separately registered PySR route broadened the hypothesis space; the authors curated, implemented and precomputed the resulting 127 descriptor channels [21]. A later logged proposer–critic loop assembled candidates only from this registry. Deterministic code sanitized, fitted and scored every candidate, while the critic supplied advisory physics assessments. Successful and failed candidates, scores and feedback were archived. None of these routes selected terms or fitted coefficients for the reported relations.

The logged campaign used the direct OpenAI service: retained records identify gpt-5.5-2026-04-23 as a proposer and gpt-5.4-mini as a physics critic. Prompts, available logs and checksums are indexed in the Supplementary Information. Anthropic Claude Opus 4.8 participated in earlier descriptor ideation, but its access context, settings, raw transcripts and channel-level attribution were not retained; this model name is therefore author-confirmed rather than reconstructed from immutable request metadata.

Human investigators reviewed the route evidence and then fixed the geometry-only scope, mandatory $k_{\mathrm{mincut}}$, six-descriptor model size, VIF threshold, cross-validation and sign checks. After lock, deterministic TRAIN-only scripts selected the remaining terms, fitted coefficients and ran the integrity gate; no automated path connected the exploratory archive to a reported equation.

### 2.4. Protocol-Locked Fitting, Data Splits, and Integrity Checks

After protocol lock, deterministic selection started from pinned $k_{\mathrm{mincut}}$ and a 30-feature geometry whitelist on 7000 TRAIN cases. Greedy additions maximizing shuffled row-wise five-fold $R^{2}$ (random_state = 2026) were followed by one-for-one swaps subject to VIF; coefficient signs were audited, not constrained [27]. The six-term forms were frozen before 1040 TRAIN-only cases were added, and then coefficients were refitted by OLS on all 8040 TRAIN rows. Recomputed row-wise CV on this enlarged partition is a post-selection diagnostic, not nested CV. No VAL outcome entered post-lock fitting.

**Figure 2:**
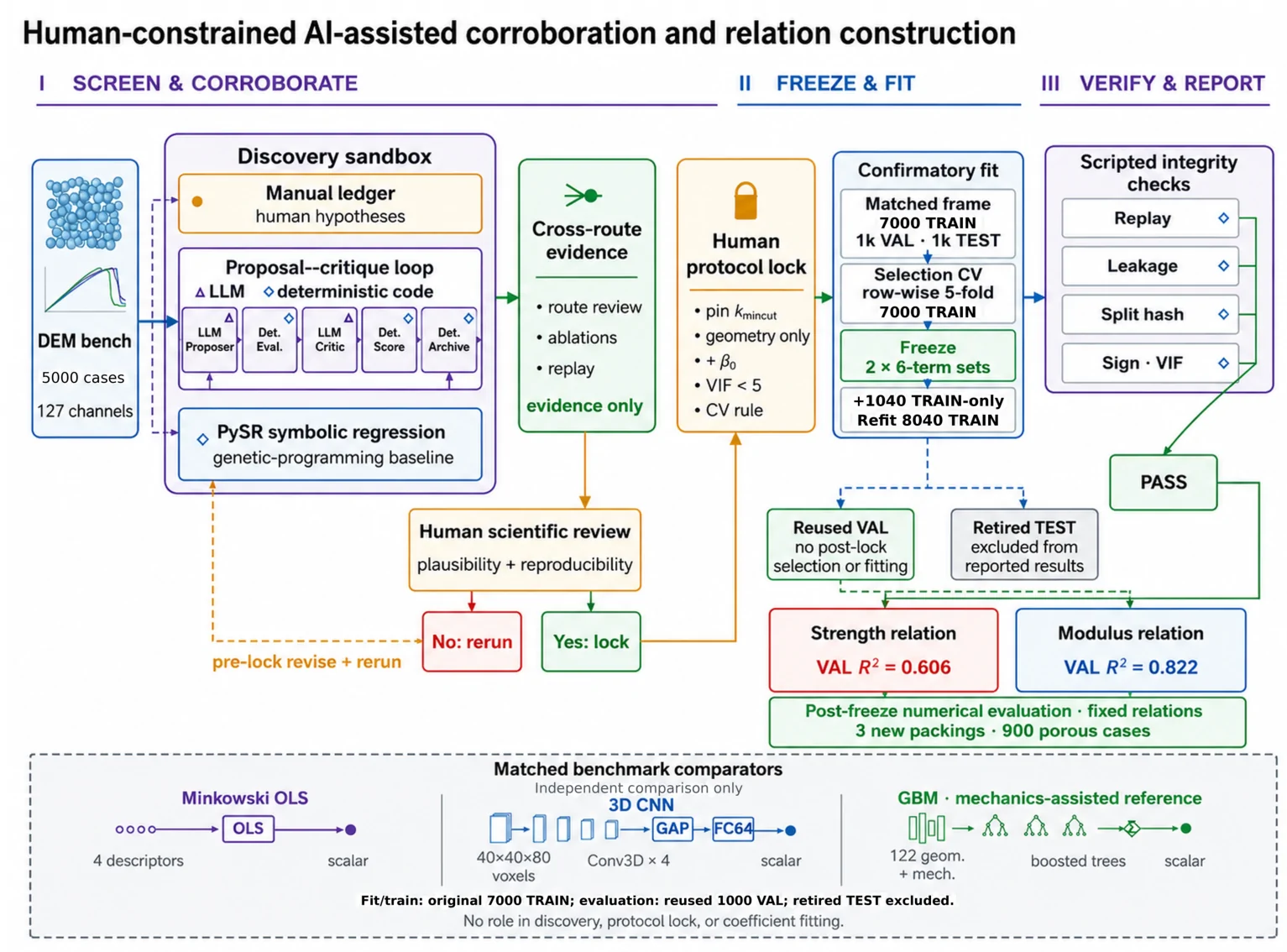
Human-constrained exploration and protocol-locked relation construction. Manual, language-model, and PySR routes were advisory; authors implemented the 127-channel registry, and human scientific review preceded protocol lock. Deterministic TRAIN-only code selected terms, refitted coefficients after the TRAIN-only top-up and alone triggered PASS. VAL is reused for reporting and TEST supplies no reported result. The post-freeze branch applies unchanged relations to three new packings. The lower band contains independently fitted comparators that supplied no descriptors, decisions or coefficients to the compact relations. Triangles denote language-model agents, blue diamonds denote deterministic code, and orange circles denote human-authored or human-decision stages. Solid arrows show the main process, whereas dashed arrows show advisory, reporting-only, or pre-lock revision paths.

VAL informed pre-lock comparisons and is therefore a reused reporting-VAL benchmark, not an independent test set. The retired 1000-case TEST partition contributes to no reported relations, headline metrics or data-bearing plots; Figure 2 shows it only as protocol context. Deterministic replay, leakage, split-hash, sign and VIF checks alone could trigger PASS; the full chronology is in the Supplementary Information.

### 2.5. Prospective Post-Freeze Numerical Evaluation Protocol

Before observing any prospective response, we froze both descriptor sets, functional forms, all coefficients and the evaluation code (canonical specification hash 43a91b8f…654e0b). The compute-bounded primary campaign changes the intact packing realization while retaining the particle-size distribution, JCFpm parameters, voxel resolution and nominal strain rate. This campaign used YADE 2026-05-27.git-814fcf6 with embedded Python 3.11.15 on the HPC system. Three new packings (P01–P03) each contribute one intact baseline and 6 families × 10 porosity levels × 5 defect seeds, for 903 cases. Each response is normalized only by the intact baseline of the same packing. No descriptor reselection, coefficient refitting, threshold tuning or retired-TEST outcome is permitted.

A separate rate-sensitivity campaign uses P01 at 0.5 and 2.0 times the nominal strain rate. Each factor contains one intact baseline and 6 families × 6 porosity levels × 2 defect seeds for 146 additional cases; factor-1 counterparts come from the primary campaign. The packing-shift and rate-perturbation results are reported separately. Preregistered execution and integrity gates, including eight analysis-excluded smoke cases, are detailed in the Supplementary Information. Primary uncertainty uses 2000 hierarchical bootstrap replicates that resample packing and then family–seed clusters; with only three packings, those intervals are necessarily imprecise. This campaign supplies prospective numerical evidence only, not experimental validation or transfer to another contact law, particle-size distribution, or voxel resolution.

### 2.6. Comparators and Matched Evaluation Frame

All comparators used the matched 7000-TRAIN/1000-VAL frame. Minkowski OLS used four conventional morphology inputs; the compact OLS relations used six descriptors plus an intercept. The PyTorch 2.7.0+cu118 3D CNN received the 40×40×80 voxel grid [28]. Four configurations were ranked on a family-stratified TRAIN-only holdout; the winning epoch count was frozen, five networks per target were refitted on all TRAIN cases, and their predictions were averaged before one reporting-VAL evaluation. The selected networks had 1,179,553 strength and 299,537 modulus weights. A histogram-gradient-boosting reference used 122 geometry-plus-mechanics inputs [27,29]; it is a high-information-accuracy reference, not a geometry-only baseline, and its modulus construction is near-target. The analysis and figure environment used Python 3.12.7, NumPy 1.26.4, pandas 2.2.2, SciPy 1.13.1, scikit-learn 1.5.1, and Matplotlib 3.9.2; the CNN used CUDA runtime 11.8.

Input dimension is distinguished from fitted model size because descriptors, voxels, regression coefficients, neural-network weights, and tree leaves are not commensurate measures. Except for the explicitly paired relation–CNN cluster bootstrap described below, between-model differences are descriptive point estimates on the same reused reporting VAL.

### 2.7. Statistical and Paired-Response Diagnostics

**Figure 3:**
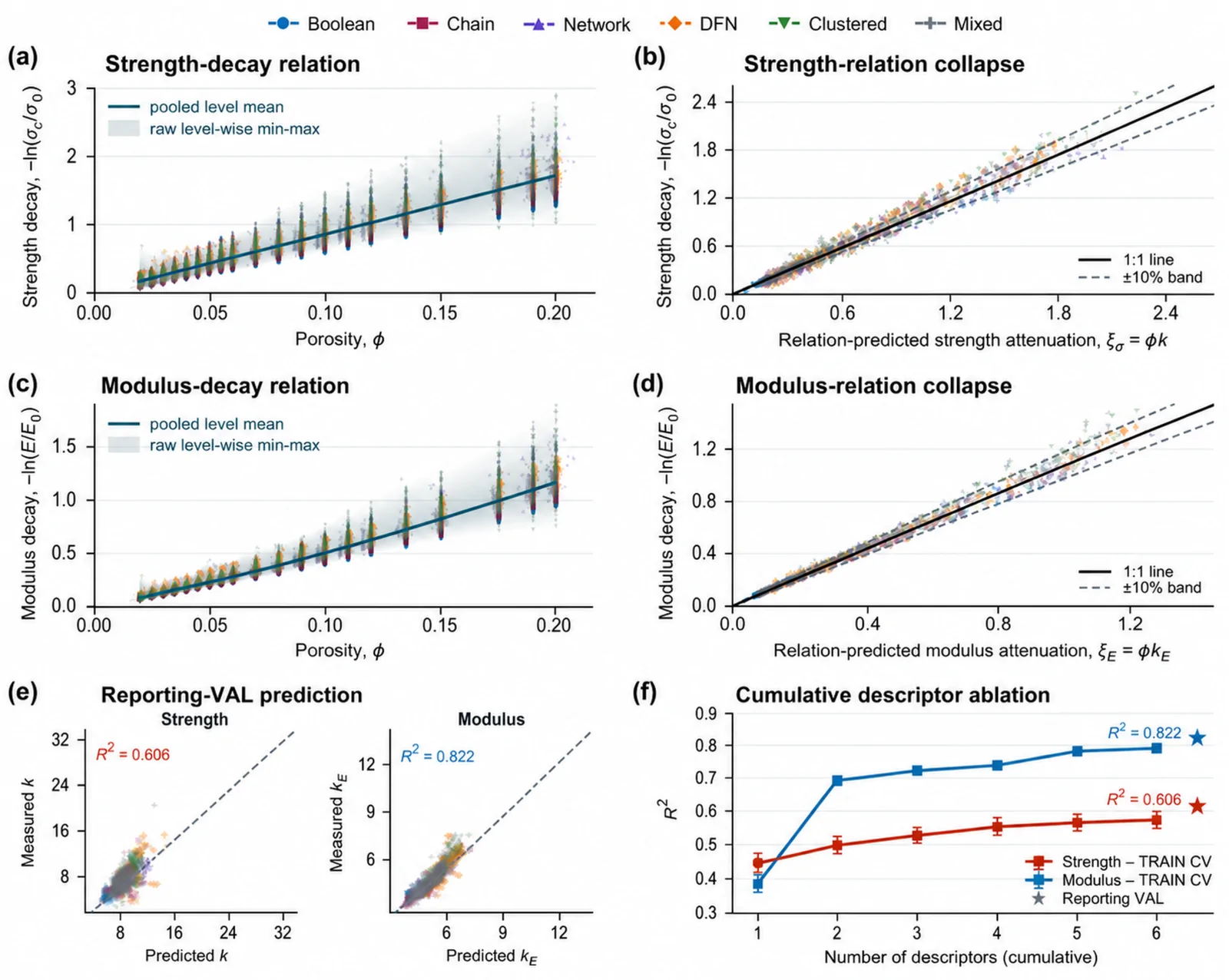
Structural-family attenuation and relation-coordinate collapse. (**a**,**c**) All 9040 non-TEST observations at stored realized porosity. The dark curve is the pooled, observation-weighted mean at each target level; the field fades to the raw single-observation minimum and maximum. It is not a family-mean envelope, density, confidence interval or prediction interval. Points were not generated or repositioned. Colors and marker shapes identify the six structural families listed in the shared legend. (**b**,**d**) Reused reporting-VAL attenuation versus fixed TRAIN-refitted coordinates $\xi_{\sigma }=\phi \hat{k}$ and $\xi_{E}=\phi {\hat{k}}_{E}$; black lines are identity and gray lines are a ±10% visual band. (**e**) Rate-scale predictions on the same reused benchmark. (**f**) Cumulative descriptors in the published order: row-wise five-fold TRAIN mean $R^{2}\pm 1$ fold population SD, with reporting-VAL stars shown separately. TEST is excluded throughout.

Performance was quantified by $R^{2}$ on *k* and $k_{E}$. Reporting-VAL intervals used 2000 fixed-model row resamples; a dependence-aware sensitivity used 2000 resamples of the 50 family–seed clusters within the family [30]. Both are conditioned on the selected specification and fitted coefficients. Figure 3f shows five row-wise TRAIN-fold means with ±1 fold population SD, not confidence intervals. None of the 500 TRAIN-target permutations matched the reporting-VAL score (plus-one p=1/501 for each target). Separate coefficient bootstraps, null distributions and VIF definitions are reported in the Supplementary Information.

For the CNN comparison, 5000 paired resamples of reporting-VAL family–seed clusters recomputed $R_{\mathrm{relation}}^{2}-R_{\mathrm{CNN}}^{2}$ from fixed predictions. This captures cluster composition, not training or selection uncertainty, and does not make VAL independent.

Because historical selection used row-wise folds, a retrospective nested family–seed-grouped audit reran the complete selector within five outer and five inner family-stratified TRAIN folds. Its outer-fold mean and population SD are the principal dependence-aware internal estimates, although they were not used historically to choose terms. A fixed-specification grouped audit on all 8040 TRAIN rows separately tests representation stability. VAL and TEST were excluded from both.

The mandatory $k_{\mathrm{mincut}}$ prior was replayed under the same nested grouped protocol as pinned, optional, or excluded. Each mode repeated selection within every outer fold and on full TRAIN; any subsequent VAL score remained reused. This is a prior-sensitivity audit, not autonomous rediscovery.

To test whether normalization left a simple porosity structure, linear, quadratic, family-only, family-plus-common-ϕ, and family-specific-ϕ ordinary-least-squares baselines were fitted on the 8040 TRAIN cases and evaluated on the unchanged reporting VAL. These are residual diagnostics on *k* and $k_{E}$, not raw-property baselines.

Three analyses are descriptive: raw family plots use 9040 non-TEST rows; reporting-family scores apply the single global fit without refitting; and a standardized ridge expands to the full tested static-geometry pool [27,31]. TRAIN-only descriptor interpretation combines marginal Spearman correlation, standardized conditional coefficients, VIF and remove-one grouped-CV loss. These conditional associations are not causal importance scores.

The paired-response analysis uses 7000 TRAIN cases and $\Delta k=k-k_{E}$. The retention illustration evaluates retention at the TRAIN medians only for interpretation; the paired-response diagnostic also shows every TRAIN case, constant-*k* contours and descriptive Gaussian-equivalent 50% covariance ellipses. These are not additional fits or confidence regions; VAL and TEST are excluded.

The held-family coefficient-portability diagnostic is a post-hoc stress test: globally fixed descriptors and form were refitted on pooled TRAIN plus VAL rows from four pure families and scored on the fifth. Mixed and TEST are excluded. Because every family informed earlier development, this is not end-to-end leave-family-out discovery, OOD or independent validation.

## 3. Results

### 3.1. Family-Structured Attenuation Remains After Porosity Normalization

Figure 3a,c plot all 9040 non-TEST specimens at their stored realized porosities. At each target level, the dark curve is the pooled observation-weighted mean, and the field fades to the raw minimum and maximum, revealing wider support at higher porosity. Family-mean rates range from 6.4005 (Boolean) to 10.1868 (Network) for strength and from 4.3021 (Boolean) to 5.7864 (DFN) for modulus.

A TRAIN-fitted ϕ-only linear baseline explained essentially none of the reporting-VAL strength-rate variation ($R^{2}=-0.003$) but 0.232 of modulus-rate variation. Allowing family-specific ϕ slopes raised performance to 0.271 and 0.536, respectively, still below the six-descriptor relations (0.606 and 0.822). Residual ϕ dependence was weak within most strength families but remained marked and family-dependent for modulus. Thus, normalization removes a leading scale factor, not all porosity dependence; topology descriptors add information beyond both porosity and family-specific slopes.

The raw design contains more observations at lower porosity while retaining specimens through the maximum realized value (ϕ=0.2079). Panels b,d map each reused reporting-VAL specimen to the fixed TRAIN-refitted coordinates $\xi_{\sigma }=\phi \hat{k}$ and $\xi_{E}=\phi {\hat{k}}_{E}$. Plotting measured attenuation against these coordinates reduces family separation around the identity line. They are empirical-relation predictions, not effective porosities; because they share the measured attenuation’s ϕ factor and use a reused benchmark, the panels are model-consistency views rather than independent validation.

### 3.2. Two Compact Relations Capture Strength and Modulus Decay

The protocol-locked TRAIN procedure produced the following strength relation:

(4) $$\begin{array}{c} k=\beta_{\sigma ,0}+\beta_{\sigma ,1}\;k_{\mathrm{mincut}}+\beta_{\sigma ,2}\;A_{\max}+\beta_{\sigma ,3}\;\rho_{\mathrm{br}}\\ \;\;\;\;\;\;\;\;\;\;\;\;\;\;+\beta_{\sigma ,4}\;\mathrm{AUC}\left[C\right]+\beta_{\sigma ,5}\;\langle |n_{z}{|\rangle }_{\mathrm{loc}}+\beta_{\sigma ,6}\;\xi_{\mathrm{clust}}, \end{array}$$

and the following modulus relation:

(5) $$\begin{array}{c} k_{E}=\beta_{E,0}+\beta_{E,1}\;k_{\mathrm{mincut}}+\beta_{E,2}\;\rho_{\mathrm{br}}+\beta_{E,3}\;A_{L}\\ \;\;\;\;+\beta_{E,4}\;{\bar{Z}}_{\mathrm{bnd}}+\beta_{E,5}\;f_{\mathrm{skel}}+\beta_{E,6}\;S_{\mathrm{nem}}. \end{array}$$

For clarity, the geometry inputs in these relations are the specimen-specific vectors

(6) $$\begin{array}{cc} g_{\sigma } & =\;(k_{\mathrm{mincut}},A_{\max},\rho_{\mathrm{br}},\mathrm{AUC}\left[C\right],\langle |n_{z}{|\rangle }_{\mathrm{loc}},\xi_{\mathrm{clust}}{)}^{T},\\ g_{E} & =\;{\left(k_{\mathrm{mincut}},\rho_{\mathrm{br}},A_{L},{\bar{Z}}_{\mathrm{bnd}},f_{\mathrm{skel}},S_{\mathrm{nem}}\right)}^{T}. \end{array}$$

Combining Equations (1) and (4) gives a direct empirical relation between the registered pore geometry and peak uniaxial compressive strength,

(7) $${\hat{\sigma }}_{c}\left(\phi ,g_{\sigma }\right)=\sigma_{c0}\exp\;\left[-\phi \left(\beta_{\sigma ,0}+\beta_{\sigma }^{T}g_{\sigma }\right)\right].$$

Here, $g_{\sigma }$ is not one universal shape factor: its six entries are calculated from each specimen’s initial three-dimensional defect field. Table 3 gives the fitted coefficients, and Supplementary Table S5 reports the observed TRAIN minimum, median and maximum of every component of $g_{\sigma }$ and $g_{E}$. Equation (7) predicts the peak compressive strength used in this study; it is not a relation for fracture toughness or an independently measured tensile fracture strength.

The relations share $\left\{k_{\mathrm{mincut}},\rho_{\mathrm{br}}\right\}$, whereas the remaining terms are target-specific. Although $k_{\mathrm{mincut}}$ was pinned by protocol, $\rho_{\mathrm{br}}$ recurred in four of five nested grouped folds for both targets. Because raw descriptor scales differ, coefficient magnitudes are not directly comparable without standardization.

Equations (4) and (5) are empirical OLS relations calibrated to the registered DEM generator, contact model, particle-size distribution and resolution. Their exponential reconstruction is physically admissible over the observed porosity range, but the coefficients are neither material constants nor evidence of a universal physical law.

Dependence-aware nested grouped CV remained positive for both targets and was lower than the historical row-wise diagnostic, particularly for strength; fixed-specification grouped CV gave the same qualitative conclusion (Table 4).

On the unchanged reused reporting-VAL benchmark, the final 8040-TRAIN refits achieved $R^{2}\left(k\right)=0.606$ and $R^{2}\left(k_{E}\right)=0.822$. Fixed-model VAL-row intervals were [0.517,0.686] and [0.797,0.842]; family–seed-cluster intervals were [0.495,0.680] and [0.787,0.849], respectively (Table 4). These intervals condition on the selected terms and fitted coefficients and do not restore independence to VAL. On the property-retention scale, the fixed relations gave $R^{2}\left(\sigma_{c}/\sigma_{c0}\right)=0.975$ and $R^{2}\left(E/E_{0}\right)=0.992$; the rate-scale results are the more demanding tests of topology.

Family-resolved evaluation identifies a response-dependent scope boundary. The modulus relation retained positive $R^{2}$ across all reporting-VAL families, whereas strength performance varied more across architectures. Complete family scores are provided in the Supplementary Information under “Final global relations by reporting family”. The pooled strength score therefore reflects substantial between-family organization, while within-family resolution is less uniform than for modulus.

The shared fitted motif does not establish unique or causal sufficiency. In particular, $k_{\mathrm{mincut}}$ reflects a protocol choice rather than independent rediscovery, while $\rho_{\mathrm{br}}$ describes branching of the pore skeleton rather than a remaining solid load-bearing skeleton. The modulus design has greater collinearity, especially in the ${\bar{Z}}_{\mathrm{bnd}}$–$f_{\mathrm{skel}}$ block. Removing $f_{\mathrm{skel}}$ lowers reporting-VAL $R^{2}\left(k_{E}\right)$ from 0.822 to 0.747 while reducing maximum VIF from 4.19 to 3.09; the block is retained, but individual negative coefficients are not given isolated causal interpretations.

TRAIN-only interpretation supports a joint rather than single-feature reading: $k_{\mathrm{mincut}}$ and $\rho_{\mathrm{br}}$ produced the largest grouped-CV losses, while reconstructed coordination was negatively associated with attenuation. The sign reversal of $f_{\mathrm{skel}}$ after adjustment indicates suppression within a correlated block rather than a causal stiffening effect. Complete statistics and bootstrap intervals are reported in the Supplementary Information.

### 3.3. Sensitivity to the Minimum-Cut Prior, Porosity Form, and Shared Scaling

When $k_{\mathrm{mincut}}$ was optional, it was selected in every outer fold and in both full-TRAIN solutions. Excluding it left grouped strength nearly unchanged (0.550±0.019 versus 0.549±0.017 when pinned) but reduced modulus from 0.773±0.024 to 0.723±0.022. The term is therefore more consequential for modulus and remains a human-admitted prior rather than an autonomous rediscovery.

A within-family grouped sensitivity held descriptor identities fixed, refitted coefficients separately in all six families and nested the stretched exponent selection. On the original retention scale, exponential $R^{2}$ ranged from 0.93495 to 0.99213 for strength and 0.98250 to 0.99800 for modulus; either power-law alternative differed from it by at most 0.00415 and 0.00462, respectively, and no form won uniformly. The exponential remains the fixed reference because neither alternative produced a consistent material gain, not because it is a uniquely identified mechanism; complete pooled and family results are in the Supplementary Information.

The common division by ϕ also creates an expected shared scale between the response rates and $k_{\mathrm{mincut}}$. Nevertheless, their TRAIN correlation remained after linear removal of porosity and family effects (r=0.476 for strength and 0.608 for modulus). In fixed family–seed-grouped CV, replacing $k_{\mathrm{mincut}}$ with the unnormalized cut attenuation reduced strength $R^{2}$ from 0.562 to 0.511 and changed modulus only from 0.785 to 0.777, while excluding the cut channel reduced them to 0.491 and 0.721. The signal is therefore not explained entirely by algebraic normalization, although the high VIF of the unnormalized replacements (4.40 for strength and 7.16 for modulus) makes the published normalized construction the better-conditioned empirical representation.

### 3.4. Compact Topology Relations Achieve Higher Accuracy than the 3D CNN

The central model comparison uses the original 9000-case frame, not the 10,040-case headline frame, so that every representation is fitted on the same 7000 TRAIN cases and evaluated on the same reused reporting VAL. On this matched frame, the four-input Minkowski baseline reached $R^{2}=0.167$ for strength and 0.382 for modulus, whereas the six-descriptor relations reached 0.603 and 0.819. The remediated five-initialization 3D-CNN ensemble, trained directly on the 40×40×80 binary voxel fields after group-separated TRAIN-only configuration selection, reached 0.428 and 0.686. Individual-run means were 0.372±0.012 and 0.649±0.046 (population SD), so ensembling gives the neural comparator a favorable evaluation. The compact relations exceeded the ensemble by 0.175 and 0.133 $R^{2}$ units; paired family–seed-cluster bootstrap intervals for these differences were [0.027,0.314] and [0.032,0.237]. These reused-benchmark intervals support a difference between the fixed predictors under the evaluated budget, not universal superiority or independent generalization.

The geometry-plus-mechanics GBM reached 0.656 for strength and 0.823 for modulus. Thus the compact relation is lower for strength and numerically just below it for modulus (0.819 versus 0.823). This reference uses mechanics-assisted inputs and is not a pure-geometry comparator.

A 60-descriptor static-geometry ridge reference reached 0.697 for strength and 0.846 for modulus on the same matched frame. This indicates that additional static geometric signal remains available, especially for strength, while placing the six-descriptor equations on an explicit parsimony–accuracy frontier rather than treating them as a predictive ceiling.

**Figure 4:**
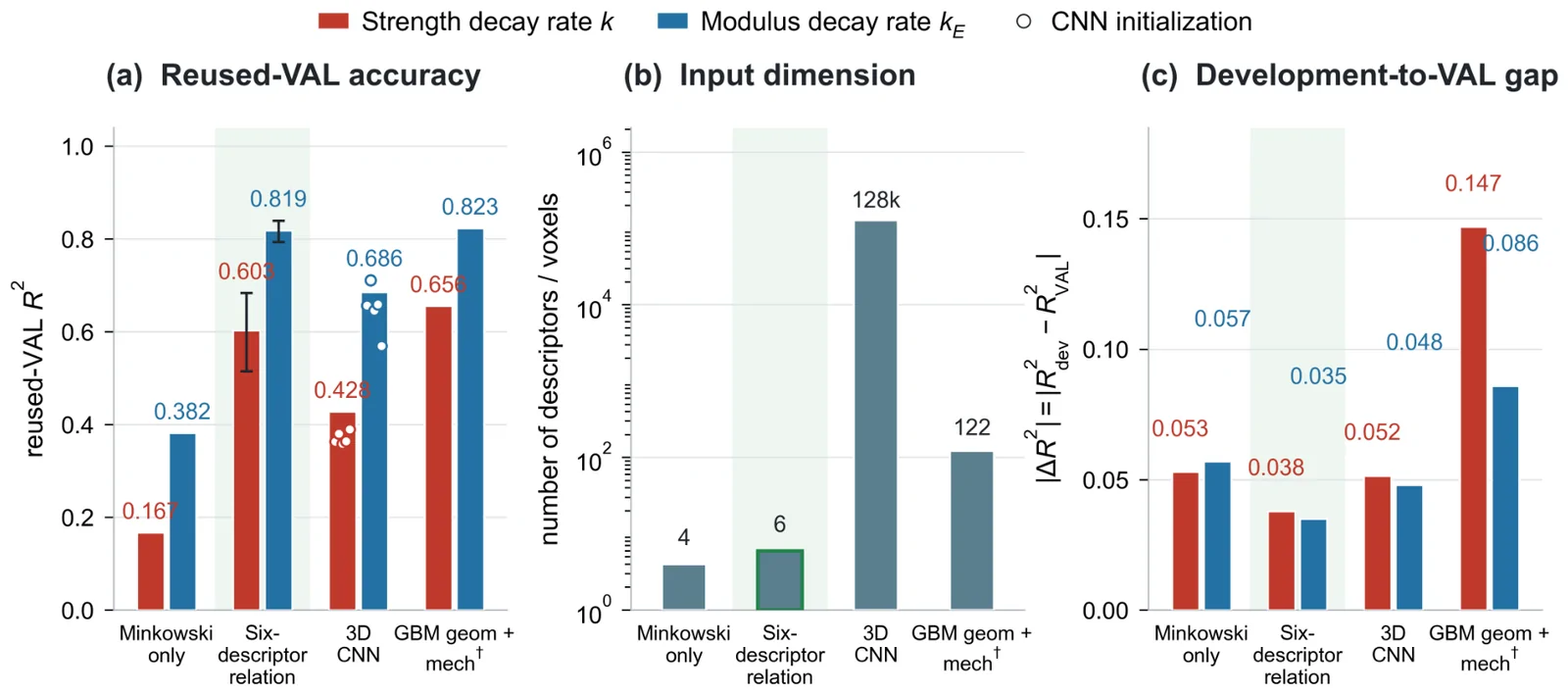
Compact representation versus the evaluated 3D CNN. All panels use matched 7000-TRAIN/1000-reused-VAL data; the TRAIN top-up and TEST are excluded. (**a**) Reporting-VAL $R^{2}$; CNN bars are five-network ensembles, circles are individual initializations, and compact-relation whiskers are fixed-model VAL-row intervals. (**b**) Preprocessed input dimension, not fitted complexity or cost. (**c**) Absolute development-to-VAL differences. Non-CNN development estimates use row-wise TRAIN CV, whereas CNN uses a grouped TRAIN holdout; the panel is therefore a provenance diagnostic, not a common generalization metric. The pale green background identifies the six-descriptor relation in each panel. The dagger (^†^) marks the mechanics-assisted GBM reference, whose modulus input is near-target.

Because Figure 4c combines row-wise CV for non-CNN models with a grouped TRAIN holdout for the CNN, it is a provenance diagnostic rather than a common generalization metric.

The TRAIN-only top-up changed reporting-VAL $R^{2}$ by only +0.003 for each target without changing VAL’s reused status.

### 3.5. Post-Freeze Evaluation Quantifies Packing-Shift Performance and Family-Specific Failures

All 1049 formal prospective DEM cases completed and passed the preregistered integrity checks. The primary analysis uses 900 porous specimens from P01–P03, each normalized by its same-packing intact baseline.

Without descriptor reselection, coefficient refitting, form changes or threshold tuning, the frozen relations achieved $R^{2}\left(k\right)=0.516$ with a 2000-replicate hierarchical 95% interval of [0.388, 0.603], and $R^{2}\left(k_{E}\right)=0.735$ with interval [0.657, 0.797] (Table 5). The corresponding attenuation-scale scores were 0.952 and 0.973, but those products retain the dominant porosity factor; the decay-rate scores remain the more stringent topology test. Packing-specific rate-scale $R^{2}$ ranged from 0.458 to 0.526 for strength and from 0.678 to 0.782 for modulus. Strength bias changed sign across packings (underprediction in P01/P02 and overprediction in P03), so the pooled result does not imply absence of packing-specific calibration shift.

The family strata expose the principal limitation rather than uniformly confirming the global strength relation. For *k*, family-specific $R^{2}$ was negative for Boolean (−0.174) and Mixed (−0.113) and ranged only from 0.082 to 0.301 in the other four families. For $k_{E}$, all six primary-family scores were positive but heterogeneous (0.221–0.800). Clustered and Network cases also showed appreciable modulus-rate underprediction. Complete packing- and family-resolved MAE, RMSE and bias values are reported in the Supplementary Information. The post-freeze evidence therefore supports more consistent packing-level transfer for modulus than for peak-strength decay, not uniform family-wise validity.

The separate P01 rate evaluation contains 144 new porous cases at 0.5 and 2.0 times the nominal rate and 72 preregistered factor-1 counterparts from the primary arm. Across the resulting 216 paired evaluation rows, pooled $R^{2}$ was 0.571 for *k* and 0.819 for $k_{E}$; factor-specific values were 0.564–0.581 and 0.818–0.819, respectively. Relative to factor 1, measured mean signed changes in *k* were −0.079 at 0.5 times and +0.163 at 2.0 times, whereas changes in $k_{E}$ were +0.003 and −0.001. Frozen geometry-only predictions are rate-invariant by construction; this comparison quantifies response sensitivity within P01 and does not show that the relations learned a rate effect. Family-resolved rate results were heterogeneous: $R^{2}\left(k\right)$ was negative for Boolean, Chain, Network and Mixed (minimum −1.403 for Chain), and $R^{2}\left(k_{E}\right)$ was negative for Network (−0.633). The pooled P01 score is therefore not a family-uniform robustness result.

## 4. Discussion

### 4.1. Representation Advantage and Paired Mechanical Interpretation

The principal result is representation-centered. The CNN had to infer connectivity, orientation, scale and bottlenecks from raw voxels, whereas the compact route encoded these quantities explicitly. After grouped TRAIN-only configuration selection, all-TRAIN refitting and five-seed ensembling, this bounded CNN remained below the compact relations on the reused benchmark, with paired cluster-bootstrap difference intervals above zero. This does not generalize to neural networks as a class. The 60-descriptor ridge and mechanics-assisted GBM retain further signal, placing the six-descriptor relations on a parsimony–accuracy frontier rather than at a predictive ceiling.

Strength and modulus share the $\left\{k_{\mathrm{mincut}},\rho_{\mathrm{br}}\right\}$ motif but retain different orientation, correlation, coordination and skeleton terms. This suggests common load-path constriction and pore-network branching, not a causal ranking: $k_{\mathrm{mincut}}$ was human-pinned, $\rho_{\mathrm{br}}$ describes the pore skeleton, and all coefficients act jointly. Strength-specific extrema, clustering and local loading-axis alignment plausibly mark localized paths that govern peak failure; modulus-specific directional order and reconstructed coordination describe distributed pre-peak load-sharing redundancy. The negative $f_{\mathrm{skel}}$ coefficient is interpreted only as conditional suppression within a correlated block. The replay shows that minimum cut is reproducibly selected when available and is especially useful for modulus, whereas correlated alternatives recover the grouped strength score.

The response contrast follows exactly from

(8) $$\Delta k=k-k_{E},\;\frac{\sigma_{c}/\sigma_{c0}}{E/E_{0}}=\exp\left(-\phi \Delta k\right).$$

Positive Δk means that strength retention decreases faster than modulus retention at the same realized porosity, as observed for 6996 of the original 7000 TRAIN specimens. The four exceptions are retained rather than edited away: one Boolean case and three Chain cases occurred at the two lowest porosity levels (ϕ≃0.020–0.025), including adjacent levels from the same Chain seed. Their raw strength-minus-modulus retention differences were only 0.0016–0.0158, which become magnified after division by small ϕ; the data do not establish that they are numerical noise. Consequently, Δk>0 is a dominant empirical pattern, not a universal sign constraint. The decomposition is an exact reparameterization of the paired responses, not a third fitted relation or a measured damage rate.

### 4.2. Response-Dependent Boundary and Falsifiable Hypothesis

**Figure 5:**
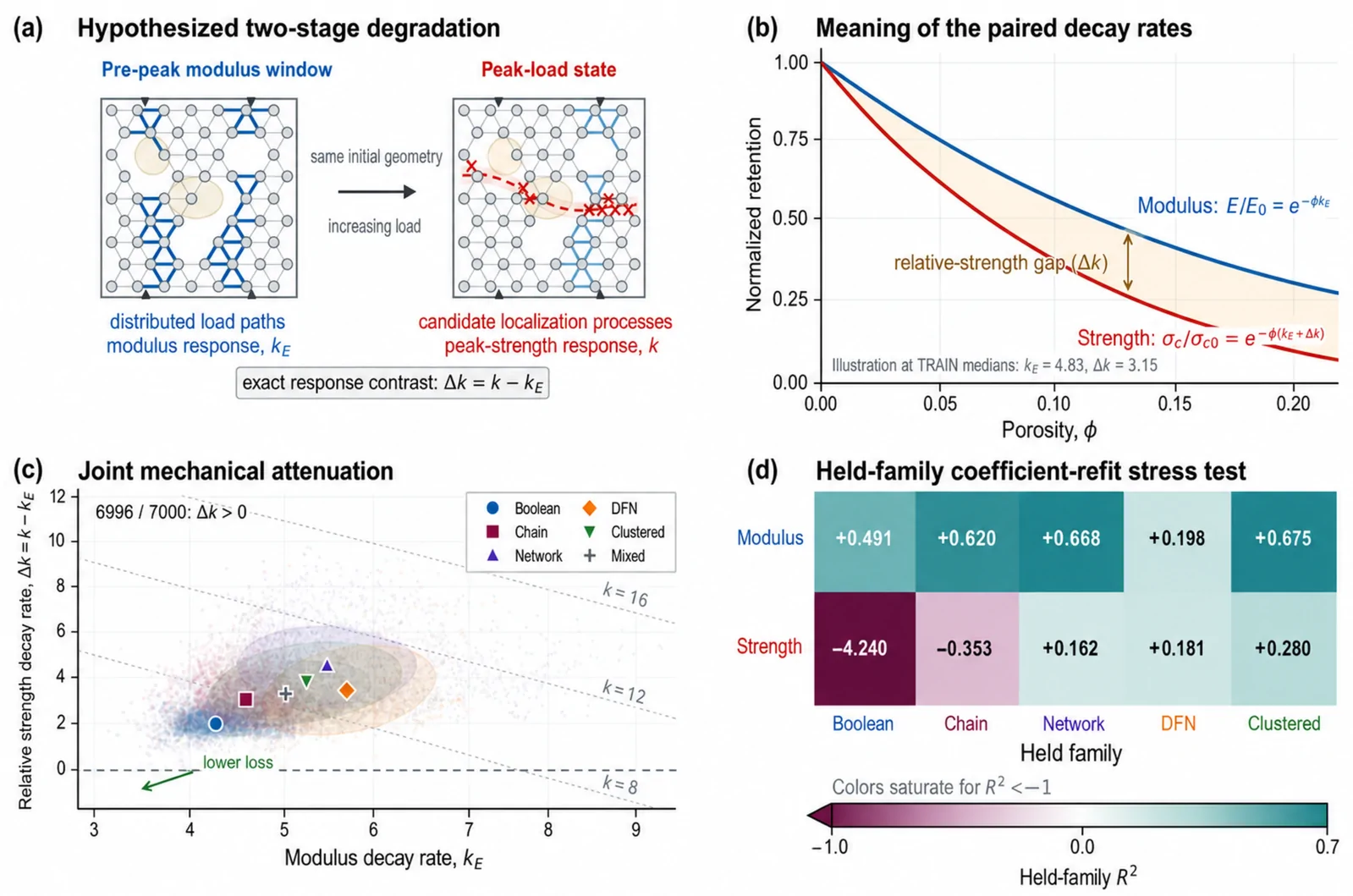
Paired attenuation and coefficient portability. (**a**) Falsifiable two-stage hypothesis; paths and damage are conceptual, not measured. (**b**) Exact retention curves at TRAIN medians $k_{E}=4.83$ and Δk=3.15; shading is illustrative and does not represent uncertainty. (**c**) TRAIN specimens; dotted lines denote constant *k* and ellipses are descriptive 50% covariance regions. Colors and marker shapes identify the six structural families listed in the panel legend. (**d**) Post-hoc held-family coefficient refits with globally fixed descriptors and form. Mixed and TEST are excluded; this is not unseen-family or independent validation. Colors saturate for $R^{2}<-1$, while cell labels retain exact values.

Figure 5a converts Δk into a testable hypothesis: initial topology may organize pre-peak load sharing, whereas bond breaking, redistribution and localization may add strength-specific loss near peak load. The data do not establish this sequence; missing static descriptors, the modulus estimator, shared 1/ϕ normalization, contact law and resolution remain alternatives. Excluding low-ϕ rows improves strength but worsens modulus prediction, confirming response-dependent normalization sensitivity.

The held-family refit gives a second boundary: coefficient portability is positive for modulus but variable for strength across pure families. Because descriptors and forms were chosen globally, this is a portability stress test, not new-family validation, and identifies strength as the harder transfer target.

A direct test should register bond breaking, failed-contact concentration, force-chain redistribution, localization and crack tortuosity at fixed loading stages. Preferential improvement of Δk and *k* over $k_{E}$ would support the hypothesis; comparable gains would favor missing static geometry or estimator effects.

### 4.3. Scientific Governance, Scope, and Validation

Language models proposed and critiqued descriptor hypotheses within a human-defined grammar; investigators fixed scope and approval rules, and deterministic scripts selected, fitted and checked the relations. Full provenance and unresolved historical metadata are reported in the Supplementary Information.

The original design fixed packing, contact law and resolution while varying family and porosity. The post-freeze campaign changed packing realization and, separately, strain rate without changing the relations. Positive pooled packing scores provide limited prospective numerical evidence, but negative strength-family scores and packing-specific bias show nonuniform portability. Contact parameters, particle-size distribution and resolution remain untested. Reporting VAL supports matched comparison, not independent validation; the prospective campaign is numerical, not experimental.

Future tests should change contact parameters, particle-size distribution, resolution and generator class under the same pre-freeze discipline. Experimental work should pair three-dimensional imaging with specimen-matched modulus, strength and damage evolution; pore graphs and orientation are image-accessible, whereas minimum-cut and regenerated-bond quantities require justified proxies.

## 5. Conclusions

Within this controlled DEM corpus, topology descriptors explained decay-rate variation beyond deletion-field porosity. A human-defined grammar and AI-assisted proposal stage produced two sparse empirical representations; deterministic code alone selected terms and fitted coefficients. On the matched reused benchmark, they outperformed conventional Minkowski inputs and the evaluated 3D-CNN ensemble while remaining inspectable. The conclusion is limited to this registered numerical comparison: physically structured geometry was a more effective compact representation than this raw-voxel model under the tested budget.

The frozen representation predicted modulus more consistently than strength, both retrospectively and across three post-freeze packings; the global strength relation failed within Boolean and Mixed strata. Together with $k=k_{E}+\Delta k$, this motivates—but does not establish—a two-stage hypothesis in which initial topology organizes pre-peak load sharing and evolving damage adds peak-strength loss. Nonpositive Δk cases, form/prior sensitivities and family heterogeneity preclude universality. The evidence is numerical; contact-law, particle-size, resolution, generator-class and experimental transfer tests remain necessary.

## Figures and Tables

**Table 1:** Data-ledger roles. Reporting VAL informed development before protocol lock and is therefore reused rather than independent; TEST is retired from all reported evidence.

Ledger	TRAIN	Reporting VAL	Retired TEST	Role in This Study
Early bench	3000	1000	1000	Descriptor construction and exploratory route diagnostics
Matched frame	7000	1000	1000	Term selection and matched Minkowski/CNN/GBM comparisons
Final ledger	8040	1000	1000	Frozen-form coefficient refit and reused-benchmark reporting

**Table 2:** Physics-organized descriptors retained in the compact degradation relations. Grouped rows follow the physical hierarchy used to interpret the grammar-constrained AI outputs. Compact definitions expose the representation, operation and summary underlying each scalar; the construction column states the geometric information supplied to the final regression. The table contains the ten unique retained descriptors rather than the complete 31-column protocol-locked pool. The Supplementary Information enumerates the full pool and gives exact implementation details for the retained descriptors.

Physical Level	Descriptor	Compact Definition	Construction and Mechanical Content	Relation
Load-path bottleneck	$k_{\mathrm{mincut}}$	$-\phi^{-1}\ln{\tilde{C}}_{\mathrm{mincut}}$	Loss of normalized top-to-bottom minimum-cut capacity in the reconstructed initial cohesive graph; measures axial load-path constriction.	Both
Connectivity and pore skeleton	$\rho_{\mathrm{br}}$	$N_{\mathrm{br}}/L_{\mathrm{skel}}$	Density of pore-skeleton voxels with at least three neighbors; measures ramification of the defect network.	Both
	$f_{\mathrm{skel}}$	$L_{\mathrm{skel}}/N_{\mathrm{pore}}$	Skeleton length per pore voxel; characterizes how extensively the pore phase is organized into connected medial paths.	Modulus
Spatial organization and correlation	AUC[C]	${\langle S_{2}\left(r\right)/\phi^{2}-1\rangle }_{r}$	Radially averaged excess two-point correlation over the registered range; measures non-random spatial organization across scales.	Strength
	$\xi_{\mathrm{clust}}$	$\min\{r/r_{\max}:C\left(r\right)\leq 0\}$	First normalized zero crossing of the excess correlation; measures the characteristic clustering extent.	Strength
Orientation and anisotropy	$A_{\max}$	$\max_{\ell }\left(1-\lambda_{3}/\lambda_{1}\right)$	Maximum multiscale anisotropy of the trace-normalized interface-gradient structure tensor.	Strength
	$\langle |n_{z}{|\rangle }_{\mathrm{loc}}$	$\langle |n_{z}{|\rangle }_{\mathrm{loc}}$	Planarity-weighted local alignment of the pore geometry with the loading axis.	Strength
	$A_{L}$	${\langle Q_{zz}\rangle }_{\ell }$	Multiscale loading-axis component of the normalized interface tensor; isolates load-oriented anisotropy.	Modulus
	$S_{\mathrm{nem}}$	${\langle \left(3\lambda_{1}-1\right)/2\rangle }_{\ell }$	Multiscale nematic order of the interface tensor; measures coherent directional organization.	Modulus
Solid-bearing architecture	${\bar{Z}}_{\mathrm{bnd}}$	$N^{-1}\sum_{i}Z_{i}^{\mathrm{alive}}$	Mean alive coordination in the geometry-reconstructed initial cohesive graph; describes local load-sharing redundancy.	Modulus

${\tilde{C}}_{\mathrm{mincut}}=C_{\mathrm{mincut}}/C_{\mathrm{mincut},0}$ after the registered numerical clipping. Tensor eigenvalues are ordered $\lambda_{1}\geq \lambda_{2}\geq \lambda_{3}$; *ℓ* indexes the registered Gaussian scales. “Both” denotes descriptors retained in both response relations, not identical fitted coefficients.

**Table 3:** Fitted coefficients of Equations (4) and (5). The coefficient in each row multiplies the listed term; j=0 denotes the intercept. Values are from the final ordinary-least-squares refit on 8040 TRAIN cases.

Relation	Coefficient	Multiplied Term	Estimate
*k*	$\beta_{\sigma ,0}$	1	0.8529
	$\beta_{\sigma ,1}$	$k_{\mathrm{mincut}}$	0.5008
	$\beta_{\sigma ,2}$	$A_{\max}$	2.1087
	$\beta_{\sigma ,3}$	$\rho_{\mathrm{br}}$	2.9905
	$\beta_{\sigma ,4}$	AUC[C]	0.7049
	$\beta_{\sigma ,5}$	$\langle |n_{z}{|\rangle }_{\mathrm{loc}}$	4.3107
	$\beta_{\sigma ,6}$	$\xi_{\mathrm{clust}}$	0.8889
$k_{E}$	$\beta_{E,0}$	1	6.195
	$\beta_{E,1}$	$k_{\mathrm{mincut}}$	0.1953
	$\beta_{E,2}$	$\rho_{\mathrm{br}}$	3.0097
	$\beta_{E,3}$	$A_{L}$	5.7505
	$\beta_{E,4}$	${\bar{Z}}_{\mathrm{bnd}}$	−0.6411
	$\beta_{E,5}$	$f_{\mathrm{skel}}$	−9.6168
	$\beta_{E,6}$	$S_{\mathrm{nem}}$	1.9412

**Table 4:** Dependence-aware and reporting performance of the final relations. Nested grouped values repeat selection within five outer family–seed-grouped folds on the original 7000 TRAIN cases; fixed grouped values evaluate the frozen specification on 8040 TRAIN cases. Both are mean ± fold population SD. Historical row-wise values are post-selection diagnostics. VAL is a reused reporting benchmark, and its cluster interval uses 2000 fixed-model family–seed resamples within family. Each relation has six descriptors and seven fitted coefficients.

Target	Nested Grouped CV	Fixed Grouped CV	Row-Wise CV	Reporting-VAL $R^{2}$	Cluster Interval	Max VIF
Strength decay rate *k*	0.549±0.017	0.562±0.054	0.574	0.606	[0.495, 0.680]	1.89
Modulus decay rate $k_{E}$	0.773±0.024	0.785±0.014	0.786	0.822	[0.787, 0.849]	4.19

**Table 5:** Prospective numerical performance on 900 porous specimens from three post-freeze packing realizations. Bias is predicted minus measured. Intervals are 2000-replicate hierarchical percentile intervals obtained by resampling packing and then family–seed clusters; with only three packings, they have limited packing-level precision. No interval is an experimental confidence statement.

Outcome	$R^{2}$	95% Interval	MAE	RMSE	Bias
Strength-decay rate *k*	0.516	[0.388, 0.603]	0.984	1.323	−0.284
Modulus-decay rate $k_{E}$	0.735	[0.657, 0.797]	0.328	0.471	−0.109
Strength decay $-\ln(\sigma_{c}/\sigma_{c0})$	0.952	[0.940, 0.963]	0.084	0.121	−0.029
Modulus decay $-\ln(E/E_{0})$	0.973	[0.961, 0.983]	0.034	0.064	−0.014

## Data Availability

The original contributions presented in this study are included in the article/Supplementary Materials. Further inquiries can be directed to the corresponding author.

## Supplementary Materials

The following supporting information can be downloaded at https://www.mdpi.com/article/10.3390/ma19173756/s1. Supplementary File S1: Supplementary Information reports simulation settings, the partition ledger, descriptor definitions, fitting and comparator protocols, uncertainty analyses and extended diagnostics. Supplementary File S2: Supplementary Data Archive provides processed data, frozen specifications, manifests, logs, checksums, analysis code and available plotting-source snapshots. Historical request-level AI replay, original voxel fields and bitwise regeneration of the original DEM corpus are outside its scope.
