# Supplementary material for: Human-Constrained AI-Assisted Identification of Defect-Topology Relations for Mechanical Degradation in Porous Solids: A Discrete-Element Study
Source: Materials (Basel). 2026 Sep 3;19(17):3756. doi: 10.3390/ma19173756 (PMC13567631; doi:10.3390/ma19173756)
Supplement: Supplementary file 1 [file materials-19-03756-s001.zip › File S1. Supplementary_Information.pdf]

Supplementary Information

Human-Constrained AI-Assisted Identification of Defect-Topology Relations for Mechanical Degradation in Porous Solids: A Discrete-Element Study

Yi Zhang[1], Yifei Liu[1,*] and Heping Xie[1]

[1] Institute of Deep Earth Sciences and Green Energy, College of Civil and Transportation Engineering, Shenzhen University, Shenzhen 518060, China

[*] Correspondence: yfliu@szu.edu.cn (Y.L.)

Abstract

This Supplementary Information records the implemented simulation, data-partition, descriptor, advisory-search, protocol-locked fitting, comparator, uncertainty, and post-freeze evaluation procedures supporting the main manuscript. It is intentionally explicit about provenance boundaries. The reported empirical relations are ordinary-least-squares fits obtained after a human protocol lock; manual enumeration and a language-model loop supplied historical advisory exploration, while PySR remains a legacy/preregistered comparison route without a complete matched-result bundle. None determined the published terms or coefficients. The 1,000-case VAL partition is a reused reporting benchmark, not a fresh confirmation set. The 1,000-case TEST partition is retired and is excluded from every reported dual-relation metric. A historical search-time held-family helper nevertheless included TEST-labelled rows in one reward component; the defect, repair, and candidate replay are disclosed in section S5.3. A separate 1,049-case post-freeze DEM campaign supplies prospective numerical evidence under the unchanged specification. All evidence remains synthetic JCFpm discrete-element evidence rather than experimental validation.

S1 Scope, terminology, and evidence status

Throughout this document, k denotes the *strength decay rate* and k_E denotes the *modulus decay rate*. The word “modulus” is used for the mechanical target; “stiffness” appears only when it is part of an implemented contact-model or time-step parameter. The evidence universes are kept separate:

- the master corpus contains 10,040 cases: 8,040 TRAIN, 1,000 reused reporting VAL, and 1,000 retired TEST;
- the non-TEST descriptive pool contains 9,040 TRAIN+VAL cases;
- the matched comparator frame contains the original 7,000 TRAIN, the same 1,000 reporting VAL, and 1,000 retired TEST cases, of which only TRAIN and VAL enter the reported comparison;
- the discovery bench contains the original 5,000 cases and 127 exploratory channels.

The distinction matters because the final coefficient refit uses 8,040 TRAIN cases, whereas the four-model comparison uses the original 7,000-case TRAIN frame. No quantitative real-rock or experimental dataset is used as external validation in the five-figure study.

S2 Virtual specimens and discrete-element protocol

S2.1 Frozen packing and JCFpm parameters

The virtual specimens were generated in YADE using its jointed cohesive-frictional particle model (JCFpm) [1, 2]. Before bond rupture, eligible near-neighbour contacts transmit normal and tangential forces through cohesive interactions. A contact bond may fail in tension or shear according to its contact-scale tensile and cohesion parameters; after failure, the contact can transmit compressive and frictional forces but no longer the intact cohesive bond. Consequently, the 50-MPa tensile parameter and 250-MPa cohesion parameter in table S1 are bond/contact thresholds, not specimen-scale uniaxial compressive strength or macroscopic cohesion. The input friction angle is likewise the contact Coulomb parameter, with $\mu = \tan 35°$; no macroscopic Mohr–Coulomb friction angle was independently calibrated.

Every original porous case inherited one frozen cuboidal matrix packing and one loading protocol. This controlled design removes packing-to-packing variability from comparisons among defect architectures, but it

also prevents the original corpus from estimating transfer across particle realizations. The prescribed distribution was $R \sim \mathcal{U}(0.20, 0.30)$ mm, with mean $\bar{R} = 0.25$ mm and

$$\mathrm{CV}(R) = \frac{0.30 - 0.20}{\sqrt{12}\,(0.25)} = 0.115. \tag{S1}$$

The 0.50-mm mean diameter gives approximately 20 mean particle diameters across the 10-mm width and 40 across the 20-mm height. The 0.10-mm span provides modest polydispersity to suppress equal-sphere ordering without introducing a distinct second size population. This was a resolution–cost choice for the 10,040-case sweep, not a particle-size convergence result. No particle-resolution study was performed, so coefficients are restricted to this discretization until prospectively tested. During Phase 0 the 50-MPa contact tensile threshold and 250-MPa shear-cohesion parameter were adjusted together at a fixed 1:5 ratio against a numerical pore-free UCS acceptance range of 60–150 MPa. The retained pore-free UCS was 84.3464 MPa. This contact-parameter calibration did not use, or establish, a macroscopic tensile strength.

Table S1: Frozen packing, material, loading, and numerical settings. Values are implementation settings, not fitted relation parameters.

Quantity	Implemented value
Specimen domain	$10 \times 10 \times 20$ mm^3; loading along z
Number of spheres	14,800
Particle-radius distribution	Uniform over 0.20–0.30 mm
Realized particle radius	Mean 0.249689 mm; standard deviation 0.028861 mm
Realized particle-radius range	0.200075–0.299975 mm
Packing seed	42
Realized solid packing fraction	0.501878
Particle density	2500 $\mathrm{kg\,m^{-3}}$
Particle-scale Young's modulus	130 GPa
Tangential-to-normal contact-stiffness parameter	0.3
Friction angle	35°
JCFpm tensile strength	50 MPa
JCFpm cohesion	250 MPa
Interaction-radius multiplier	1.1
Loading engine	YADE `UniaxialStrainer`
Nominal axial strain rate	-0.5 s^{-1}
End-particle sets	Negative and positive loading-axis sets returned by `uniaxialTestFeatures()`
Strainer constraints	`asymmetry=0`, `blockDisplacements=1`, `blockRotations=1`, `setSpeeds=0`
Macroscopic stress area	Bounding-box cross-sectional area returned by `uniaxialTestFeatures()` (nominally 100 mm^2)
Stopping strain	Magnitude 0.003
Damping coefficient	0.4
Global-stiffness time-step safety factor	0.5
Maximum iteration count	300,000
Stress-recording interval	Every 250 iterations
Voxel grid	$40 \times 40 \times 80$ Boolean grid
Voxel edge length	0.25 mm

The matrix-only reference simulation gave

$$E_0 = 38.6601 \text{ GPa}, \qquad \sigma_{c0} = 84.3464 \text{ MPa}. \tag{S2}$$

For each porous case, σ_c was the peak compressive stress. The modulus E was estimated by ordinary least squares over pre-peak samples between 30% and 50% of σ_c. A secant estimate was used only when fewer than five samples lay in this window. All response ratios use the matrix-only reference generated by the same numerical pipeline.

Macroscopic fracture toughness K_{IC} was neither prescribed nor calibrated. Although the bonded-contact law generates progressive rupture and crack-like localization, its contact tensile threshold cannot be relabelled as a specimen-scale fracture toughness. A future calibration would require an independently simulated and experimentally matched notched-specimen geometry, crack-length convention and energy- or stress-intensity-based evaluation.

S2.2 Target defect porosity and voxel-to-particle mapping

The 20 prescribed target porosities were

$$0.020,\ 0.025,\ 0.030,\ 0.035,\ 0.040,\ 0.045,\ 0.050,\ 0.055,\ 0.060,\ 0.070,$$
$$0.080,\ 0.090,\ 0.100,\ 0.110,\ 0.120,\ 0.135,\ 0.150,\ 0.175,\ 0.190,\ 0.200.$$

Defect porosity ϕ was defined as the ratio of the volume of the registered deletion region, within which particles were removed, to the nominal specimen volume; hereafter, it is termed porosity. A particle was removed when its centre lay in a defect voxel, and the deletion-region volume was obtained from the registered defect voxels. Thus ϕ excludes the geometric interparticle void fraction ϕ_{ip} of the granular packing. In the original controlled corpus ϕ_{ip} is fixed because every case shares the same intact packing; therefore its separate mechanical effect is not identifiable. We neither add ϕ and ϕ_{ip} nor call their sum a measured total porosity, because deleted voxels and granular voids require an explicit union-volume convention to avoid overlap. All analysis used realized defect porosity rather than the nominal target. The surviving initial contact graph was then cohered and loaded under the common JCFpm protocol. The design targeted $\phi_{\mathrm{target}} \leq 0.20$; the largest realized defect porosity in the stored corpus is 0.207890625.

S2.3 Six defect-family generators

Table S2: Frozen defect-family generators and recorded parameters. Analysis used the resulting realized porosity rather than the nominal target.

Family	Registered construction and fixed parameters
Boolean	Independent uniformly placed pore primitives; overlap allowed. Primitive radius 0.4 mm.
Chain	Eight persistent random-walk traces; case-seeded isotropic initial directions; inter-pore spacing 0.4 mm; persistence 0.98; primitive radius 0.4 mm.
Network	Random geometric graph with 50 nodes; connection radius 6.5 mm; edge spacing 0.4 mm; node-radius multiplier 1.5; primitive radius 0.4 mm.
DFN	Pure discrete-fracture-network family formed from isotropically oriented circular disks; disk radius 1.8 mm; finite numerical thickness 0.5 mm (two voxels). These are crack-like deletion zones, not zero-aperture discontinuities or frictional crack surfaces; the deletion construction does not resolve a specimen-scale fracture-process zone.
Clustered	Neyman–Scott construction with 15 parents; cluster radius 1.3 mm; primitive radius 0.4 mm.
Mixed	Voxel union of all five primitive families. Five mixture weights drawn from symmetric Dirichlet(2); DFN radius sampled uniformly from 1 to 3 mm; orientation concentration sampled uniformly from 0 to 0.9; preferred axis sampled as a random unit vector.

Family, target porosity, and seed determine each case. Generator parameters are therefore recoverable from the registered case identifier and seed. The common packing and voxel grid are held fixed across families.

S3 Corpus construction, partitions, and cross-validation

S3.1 Initial 5,000-case ledger and TRAIN-only extensions

The initial 5,000 cases comprised 500 cases from each of the five pure families (20 porosities times 25 seeds) and 2,500 Mixed cases (20 porosities times 125 seeds). The deterministic initial split was:

- pure families: seeds 1–15 TRAIN, 16–20 VAL, and 21–25 TEST;
- Mixed: seeds 1–75 TRAIN, 76–100 VAL, and 101–125 TEST.

This gave 3,000 TRAIN, 1,000 VAL, and 1,000 TEST cases. The first TRAIN-only extension added seeds 26–75 for Boolean, Chain, Network, and Clustered at all 20 porosities, contributing 4,000 cases. The second TRAIN-only extension added seeds 76–88 for those same four families, contributing 1,040 cases. Neither extension added DFN, Mixed, VAL, or TEST rows.

Figure 1 illustrative cases. The family response curves shown in Figure 1 use seed 42 for Boolean, Chain, Network, and Clustered and seed 1 for DFN and Mixed. They were selected as illustrative cases at comparable target porosity and are not a common-seed paired dataset or an input to any fitted relation, metric, or inferential comparison. The particle configurations and bond segments are deterministic visualizations of the corresponding DEM records.

Table S3: Final 10,040-case family and split ledger. VAL is reused for reporting; TEST is retired and excluded from the reported relations.

Family	TRAIN	Reporting VAL	Retired TEST	Total
Boolean	1,560	100	100	1,760
Chain	1,560	100	100	1,760
Network	1,560	100	100	1,760
DFN	300	100	100	500
Clustered	1,560	100	100	1,760
Mixed	1,500	500	500	2,500
Total	8,040	1,000	1,000	10,040

S3.2 Selection-stage and refit-stage distinction

The six descriptors for each relation were selected using the original 7,000-case TRAIN partition. After the descriptor sets were frozen, ordinary-least-squares coefficients, including an unconstrained intercept, were refitted on all 8,040 TRAIN cases. The unchanged 1,000-case VAL set is reported as a reused benchmark. The two reported relations were not evaluated on the retired 1,000-case TEST partition.

Each family–generator-seed group contributes exactly 20 porosity levels. The final ledger contains 402 such groups in TRAIN, 50 in VAL, and 50 in TEST, with no group identifier shared across partitions. The original locked selector used shuffled row-wise `KFold` with `random_state=2026`; therefore rows from one TRAIN group could occur in different folds. Descriptor selection used this five-fold score on the original 7,000 TRAIN rows, and the historical fixed-specification diagnostic was recomputed on all 8,040 TRAIN rows. These row-wise values are retained for provenance but are not the dependence-aware headline sensitivity.

Two retrospective group-aware audits were subsequently run without accessing VAL or TEST outcomes. First, the fixed published specifications were evaluated by five-fold `StratifiedGroupKFold` on all 8,040 TRAIN rows, using family–seed as the group and family as the stratification label. Second, nested grouped cross-validation was run on the original 7,000 TRAIN rows (350 groups): each of five outer folds repeated the complete six-term greedy-addition and one-for-one-swap selection inside a five-fold grouped inner loop, with k_{mincut} pinned and the VIF threshold retained. Both levels used shuffled family-stratified group folds with random state 2026. This nested result is a post-hoc robustness analysis of the registered selection procedure, not an independent external confirmation.

S3.3 Prospective post-freeze numerical campaign

Before generating any new mechanical response, the two descriptor sets, exponential forms, 14 OLS coefficients, preprocessing conventions and evaluation code were serialized under canonical specification SHA-256 `43a91b8fa19c4cdfdaedc38e5d990753f2eed480949306135fc7d6fa34654e0b`. The primary campaign changes only the intact particle realization: packing seeds 104729 (P01), 130363 (P02) and 169087 (P03) retain the registered particle-size distribution, contact law, voxel grid and nominal loading rate. For each packing, one intact baseline normalizes its porous descendants. The formal primary design contains $3 \times 6 \times 10 \times 5 = 900$ porous cases plus three baselines, using target porosities 0.02, 0.03, 0.04, 0.06, 0.08, 0.10, 0.12, 0.15, 0.175 and 0.20 and defect seeds 1001–1005.

A separate rate-sensitivity design uses P01 at 0.5 and 2.0 times the nominal rate. Each factor contains $6 \times 6 \times 2 = 72$ porous cases plus one intact baseline, at target porosities 0.02, 0.04, 0.08, 0.10, 0.15 and 0.20 and seeds 1001–1002. Factor-1 counterparts come from the primary campaign, giving 146 newly simulated rate-sensitivity cases and 1,049 formal new cases in total. Eight production-shaped smoke cases are a computational gate and are excluded from analysis.

The post-freeze evaluator prohibits descriptor reselection, coefficient refitting, form changes, threshold tuning and use of retired TEST. Every completed case must have an atomic completion record, matching hashes, finite positive strength and modulus, at least 20 stress–strain samples, adequate terminal strain and the correct same-packing intact baseline. All submitted cases, including simulation or quality-control failures, remain in the outcome ledger. Primary uncertainty is a 2,000-replicate hierarchical bootstrap that resamples packing and then family–seed clusters; packing-specific scores and the fraction of invalid cases are reported alongside the pooled result. With only three new packings, the interval is a deliberately limited numerical transfer assessment rather than experimental or broad external validation.

S4 Mechanical targets and registered descriptor mapping

S4.1 Porosity-scaled decay rates

The specimen targets were

$$k = -\frac{\ln(\sigma_c/\sigma_{c0})}{\phi}, \qquad k_E = -\frac{\ln(E/E_0)}{\phi}. \tag{S3}$$

Equivalently,

$$\frac{\sigma_c}{\sigma_{c0}} = \exp(-\phi k), \qquad \frac{E}{E_0} = \exp(-\phi k_E). \tag{S4}$$

All stored source response ratios were positive. The 1,040-case top-up assembly applied a numerical lower bound of 10^{-9} before taking logarithms; this bound was inactive for those rows.

S4.2 Publication symbols, archive fields, and definitions

Table S4: Registered descriptors in the reported relations. Raw coefficients apply to these exact implementations and numerical scales.

Target	Symbol	Repository field	Implemented definition and qualification
Both	k_{mincut}	`k_mincut`	Porosity-scaled loss of normalized top-to-bottom minimum-cut capacity in the reconstructed initial cohesive graph; see equation (S5).
Both	ρ_{br}	`branch_den`	Number of pore-skeleton voxels with at least three neighbours in a $3 \times 3 \times 3$ neighbourhood, divided by total skeleton length in voxels.
Strength	$A_{\max}$	`dg_Amax`	Maximum over Gaussian scales $\{1, 2, 3, 4\}$ voxels of $1 - \lambda_3/\lambda_1$ for the trace-normalized, interface-gradient structure tensor.
Strength	$\mathrm{AUC}[C]$	`dg_aucC`	Mean radial excess two-point correlation $C(r) = S_2(r)/\phi^2 - 1$ over integer radii from one voxel to half the smallest grid dimension. This is a voxel-field correlation, not a pore-centre pair correlation.
Strength	$\langle \lvert n_z \rvert \rangle_{\mathrm{loc}}$	`loc_normal_z`	Planarity-weighted mean absolute z component of the smallest-variance local covariance eigenvector. Radius-three-voxel windows and at most 300 deterministically sampled pore voxels per case were used.
Strength	ξ_{clust}	`dg_xiclust`	First normalized radius at which $C(r) \le 0$; set to one if no crossing occurs in the implemented range.
Modulus	A_L	`dg_AL`	Multiscale average of the loading-axis component Q_{zz} of the trace-normalized interface-gradient structure tensor.
Modulus	$\bar{Z}_{\mathrm{bnd}}$	`bnd_coord_mean`	Mean alive coordination of surviving particles in the geometry-reconstructed initial cohesive graph. It is numerically identical, row by row, to the archived as-built `coord_num` field.
Modulus	f_{skel}	`skel_frac`	Pore-skeleton length divided by pore-voxel count on the registered grid. Its coefficient is interpreted only within the correlated modulus block.
Modulus	S_{nem}	`dg_S2`	Multiscale average of $(3\lambda_1 - 1)/2$ from the same trace-normalized interface-gradient tensor. The public symbol avoids collision with the conventional two-point function $S_2(r)$; `dg_S2` is retained as the historical repository identifier.

For k_{mincut}, particles i and j were connected when their separation did not exceed $1.1(r_i + r_j)$. Edge capacity was proportional to $\pi \min(r_i, r_j)^2$. Particles in the top and bottom 1 mm grip regions were connected to source and sink nodes. If $C_{\mathrm{mincut},0}$ is the pristine-packing capacity, the descriptor is

$$k_{\mathrm{mincut}} = -\frac{\ln\bigl[\mathrm{clip}(C_{\mathrm{mincut}}/C_{\mathrm{mincut},0}, 10^{-6}, \infty)\bigr]}{\phi}. \tag{S5}$$

The correlation descriptors use a voxel-field surrogate because connected Chain, Network, and DFN structures do not admit a stable pore-centroid decomposition. The structure tensor is weighted by interface-gradient magnitude, rather than being an unweighted unit-normal tensor. These implementation choices are part of the descriptor registration and must accompany any released design matrix.

An earlier internal selection run referred to the coordination column as `coord_num`. Sample-wise verification found exactly zero difference from the geometry-reconstructed `bnd_coord_mean` column. The public equation uses $\bar{Z}_{\mathrm{bnd}}$ and `bnd_coord_mean`. This replacement does not imply that the historical selection implementation never saw the DEM-exported field name.

S4.3 Final raw-scale equations

The finalized empirical relations are

$$\begin{aligned} k = 0.8529 &+ 0.5008\, k_{\mathrm{mincut}} + 2.1087\, A_{\max} + 2.9905\, \rho_{\mathrm{br}} \\ &+ 0.7049\ \mathrm{AUC}[C] + 4.3107\, \langle |n_z| \rangle_{\mathrm{loc}} + 0.8889\, \xi_{\mathrm{clust}}, \end{aligned} \tag{S6}$$

and

$$\begin{aligned} k_E = 6.1950 &+ 0.1953\, k_{\mathrm{mincut}} + 3.0097\, \rho_{\mathrm{br}} + 5.7505\, A_L \\ &- 0.6411\, \bar{Z}_{\mathrm{bnd}} - 9.6168\, f_{\mathrm{skel}} + 1.9412\, S_{\mathrm{nem}}. \end{aligned} \tag{S7}$$

Each equation has six descriptors plus an intercept, hence seven fitted coefficients. The two specifications share $\{k_{\mathrm{mincut}}, \rho_{\mathrm{br}}\}$ as a fitted motif. The minimum-cut term was fixed a priori by protocol, whereas pore-skeleton branch density recurred under the two target-specific selections. The latter measures ramification of the pore skeleton, not branching of the remaining solid skeleton. The coefficients act jointly on unlike raw descriptor scales; they are not a causal ranking of isolated mechanisms. In particular, the maximum VIF of the modulus design is 4.19, so the negative f_{skel} coefficient is interpreted as part of a correlated block rather than a universal marginal effect.

For explicit mapping from pore geometry to the mechanical responses, define

$$\mathbf{g}_\sigma = (k_{\mathrm{mincut}}, A_{\max}, \rho_{\mathrm{br}}, \mathrm{AUC}[C], \langle |n_z| \rangle_{\mathrm{loc}}, \xi_{\mathrm{clust}})^{\mathsf{T}}, \qquad \mathbf{g}_E = (k_{\mathrm{mincut}}, \rho_{\mathrm{br}}, A_L, \bar{Z}_{\mathrm{bnd}}, f_{\mathrm{skel}}, S_{\mathrm{nem}})^{\mathsf{T}}. \tag{S8}$$

The strength relation is therefore

$$\widehat{\sigma}_c(\phi, \mathbf{g}_\sigma) = \sigma_{c0} \exp\bigl[-\phi\, (\beta_{\sigma,0} + \boldsymbol{\beta}_\sigma^{\mathsf{T}} \mathbf{g}_\sigma)\bigr], \tag{S9}$$

with the coefficient values given in the main text. This is a relation for peak uniaxial compressive strength, not fracture toughness or tensile fracture strength. The vectors are evaluated separately for every specimen rather than assigned one family-level value. Their observed TRAIN distributions are summarized in table S5; reporting VAL and retired TEST are excluded from these ranges.

S5 Advisory discovery routes and human protocol lock

S5.1 Status of the exploratory routes

The 127-channel exploratory bench was created in a grammar-constrained, AI-assisted descriptor-ideation stage followed by author curation and implementation. Human investigators defined

$$f = \mathcal{S} \circ \mathcal{O} \circ \mathcal{R}(\mathcal{G}),$$

where $\mathcal{G}$ denotes the initial three-dimensional defect geometry, $\mathcal{R}$ maps that geometry to fields, interfaces, skeletons, or particle–bond graphs, $\mathcal{O}$ applies admissible multiscale, tensor, connectivity, or bottleneck operations, and $\mathcal{S}$ returns scalar summaries. The information boundary admitted initial geometry and loading direction but excluded mechanical outcomes. Within this human-defined grammar, language models assisted candidate mathematical and physical ideation. Human investigators curated, implemented and precomputed the 127

Table S5: Observed values of the geometry-descriptor vectors in the 8,040-case TRAIN ledger. Entries are reported on the raw scales used by the fitted equations.

Vector	Component	Minimum	Median	Maximum
$\mathbf{g}_\sigma, \mathbf{g}_E$	k_{mincut}	2.1396	4.8782	15.3937
$\mathbf{g}_\sigma$	$A_{\max}$	0.06967	0.33171	0.75427
$\mathbf{g}_\sigma, \mathbf{g}_E$	ρ_{br}	0	0.20668	0.82647
$\mathbf{g}_\sigma$	AUC$[C]$	0.09060	0.95756	4.50068
$\mathbf{g}_\sigma$	$\langle \lvert n_z \rvert \rangle_{\mathrm{loc}}$	0.21046	0.48444	0.83357
$\mathbf{g}_\sigma$	ξ_{clust}	0.15	1.00	1.00
$\mathbf{g}_E$	A_L	0.23022	0.33654	0.54144
$\mathbf{g}_E$	$\bar{Z}_{\mathrm{bnd}}$	5.4374	6.2461	6.5750
$\mathbf{g}_E$	f_{skel}	0.02239	0.10690	0.19271
$\mathbf{g}_E$	S_{nem}	0.01276	0.06764	0.38347

channels admitted to the original 5,000-case exploratory bench. A channel-level model-output-to-implementation map was not retained, so the registry is not attributed wholesale to a language model.

Manual enumeration, a later instrumented language-model loop, and a separately preregistered PySR comparison then explored this generated descriptor bank. Only legacy PySR outputs and the later protocol remain, as detailed in section S5.4. These routes shared data and descriptors; they were not independent, blind, or mutually isolated. They produced candidate concepts, feature subsets, and route-comparison evidence, but none directly selected the six reported terms or estimated the coefficients in equations (S6) and (S7).

The instrumented loop followed

$$\text{diagnostics} \to \text{Proposer} \to \text{deterministic evaluation} \to \text{Physics critic} \to \text{Score} \to \text{Archive} \to \text{Proposer}.$$

Candidate specifications were evaluated by deterministic code. The critic scored physical plausibility, parsimony, and consistency without receiving the pure-fit R^2 in its critic input. Successful and failed candidates were written to the archive, and the archive informed subsequent proposals. This later repeated campaign operated over the registered descriptor bank: execution of newly proposed AI feature code was disabled, and any new implementation would have required separate human review. This restriction separates the earlier AI-assisted ideation and human implementation stage from the audited relation-specification campaign.

The historical scorer combined clipped reporting-VAL retention, a normalized complexity penalty, physics-rule pass fraction, a normalized worst-family term, and the critic score; failed candidates received an additional fixed penalty. This implementation is retained only for provenance and replay. Because one historical worst-family helper admitted TEST-labelled rows, contaminated endpoint-ablation numbers are not promoted as clean evidence here. The scorer was not the objective used to fit either reported OLS relation.

Human review subsequently fixed the geometry-only scope, pinned k_{mincut}, limited each relation to six descriptors plus an intercept, required maximum VIF below 5, specified five-fold row-wise TRAIN cross-validation as the selection score, and required deterministic replay, leakage, split-hash, coefficient-sign, and VIF checks. The implementation exposed 30 optional candidates in addition to pinned k_{mincut} (31 available columns at search start). Starting from k_{mincut}, features were added greedily by mean cross-validated R^2, followed by local one-for-one swaps; the pinned term could not be removed. Coefficient signs were checked after the unconstrained OLS fit and were not hard constraints.

The 30 optional candidates, in implemented search order, were `layer_poro_std`, `max_layer_poro`, `min_solid_layer`, `lig_thick_mean`, `lig_thick_std`, `lig_thick_p10`, `branch_den`, `skel_frac`, `largest_frac`, `coord_num`, `surf_dens`, `spec_surf`, `max_cross_cut`, `mean_cross_cut`, `perc_z`, `perc_xy`, `loc_normal_z`, `dg_fthin3`, `dg_fthin5`, `dg_Cmax`, `dg_aucC`, `dg_xiclust`, `dg_Hmax`, `dg_aucH`, `dg_lacAUC`, `dg_lacslope`, `dg_Amax`, `dg_AL`, `dg_S2`, and `dg_aucA`. At each greedy step, the eligible candidate with maximum mean five-fold score was appended until six descriptors were present; the forward stage did not require an improvement over the incumbent, and an exact tie retained the first candidate in this order. No random restart was used. Swap search visited replaceable positions and candidates in the same deterministic order, accepted only improvements greater than 10^{-5}, selected the largest improvement (first encountered on an exact tie), and repeated until no qualifying swap remained. For each proposed set, every feature was regressed on an intercept and the remaining selected features; its VIF was $1/(1-R^2)$. The maximum VIF was rounded to two decimals before applying the strict eligibility rule VIF < 5.0.

S5.2 LLM execution provenance and stage-specific records

Execution provenance differs between the descriptor-ideation stage and the later repeated relation-specification campaign. Private working records from the repeated campaign retain role messages and identify

`gpt-5.5-2026-04-23` as the proposer and `gpt-5.4-mini` as the physics critic. The submitted archive contains sanitized reconstructed prompt templates, round-level candidate/evaluator/critic/reward/archive summaries, request code and release-time checksums; it does not expose private request transcripts. The campaign used the OpenAI Python client through the Chat Completions interface; the authors confirmed that the calls used the direct OpenAI service, although the endpoint field itself was not retained in immutable metadata. The authors confirm that Anthropic Claude Opus 4.8 participated in the earlier descriptor-ideation stage. This model name is author-confirmed, but the access context, request settings, raw stage-specific transcripts, immutable request metadata and channel-level attribution remain unavailable. Consequently, that early language-model stage cannot be replayed at request level or mapped descriptor by descriptor. Reproducibility begins with the archived author-implemented descriptor definitions and their deterministic values, followed by the protocol-locked selection and fitting stages. Author confirmation is labelled as such, and current code defaults are not substituted for missing execution metadata.

Provenance item	Status in this Supplementary Information
Provider and API endpoint	Direct OpenAI service confirmed by the authors; OpenAI Python client and Chat Completions interface recorded; endpoint field not retained
Descriptor-ideation provider/model	Anthropic Claude Opus 4.8, confirmed by the authors; access context, request settings, raw stage-specific transcripts, immutable request metadata and channel-level attribution were not retained
Repeated-campaign proposer	`gpt-5.5-2026-04-23`; reasoning effort `high`
Repeated-campaign critic	`gpt-5.4-mini`; reasoning effort `low`; used in critic-assisted runs only
Repeated comparison	Ten six-round runs on 13 June 2026: five critic-assisted and five critic-free references; one archived candidate per round, 60 candidates total. Logged timestamps span 00:14–02:15, but the timezone was not encoded.
Request controls	Initial `max_completion_tokens`=16,000 with one doubled-budget retry only after a length stop; `temperature`, `top_p`, and API seed were not supplied to these reasoning-model requests and provider defaults therefore applied.
Prompt records	Sanitized reconstructed role templates and round-level summaries are submitted; private repeated-campaign request transcripts are not included, and earlier descriptor-construction prompts were not recovered
Deterministic harness	Source-controlled and auditable through sanitized candidate, metric, critic, reward, and archive summaries
Checksum manifest	Release-time hashes of submitted AI-provenance materials are provided; unavailable historical request checksums are not inferred

The comparison used fixed data and a deterministic evaluator; the API responses themselves were not claimed to be deterministic because no provider-side seed was supplied. Every round retained proposer messages and output, sanitization notes, automatic metrics, critic messages and output where applicable, reward components, and the accumulated archive. These stage-specific qualifications prevent later campaign metadata from being silently assigned to the earlier descriptor-generation stage. They do not affect the deterministic, protocol-locked relations, whose reported terms and coefficients did not come from an agent-selected winner.

S5.3 Historical TEST-labelled held-family reward audit

The early loop documentation stated that TEST was locked because the explicit TEST accessor was not called. A later source audit found a narrower but important defect: before 15 July 2026, the evaluation side of the generic held-family helper did not filter split labels. For each pure held family it therefore evaluated all rows, including 100 TEST-labelled response rows. In the AI-feedback arm, the resulting held-family score entered the historical composite scorer; thus TEST-labelled outcomes indirectly influenced exploratory rewards even though the explicit TEST accessor remained locked.

The helper was repaired to use only TRAIN+VAL rows on the held-family evaluation side, mirroring the post-hoc diagnostic used elsewhere. All 60 archived candidates from ten six-round runs were then replayed with logged critic scores held fixed. The replay reproduced the historical logged rewards exactly under the old helper (maximum replay error 0.0). After applying the corrected helper, archive order changed in 0 of 10 runs and

the best candidate changed in 0 of 10 runs. This is a robustness audit of archived exploratory decisions, not permission to describe project-wide TEST handling as untouched.

Table S6: Historical held-family reward audit.

Audit item	Result
Source defect	Held-family evaluation side lacked a split filter
Affected content	100 TEST-labelled rows per held pure family could enter a historical held-family reward component
Explicit TEST accessor	Remained locked; this did not prevent the indirect path
Repair	Restrict held-family evaluation to TRAIN+VAL
Replay scope	60 candidates, ten runs, six rounds per run
Old-helper replay error	Maximum absolute error against logged reward = 0.0
Archive rankings changed	0/10 runs
Best candidate changed	0/10 runs
Effect on reported relations	None: agent winners did not determine the final terms, coefficients, or metrics

Historical small-sample AI-feedback-versus-greedy endpoint statistics are not reused as clean primary evidence because their search-time reward was produced before this repair. The defensible AI claim is architectural and procedural: AI-assisted descriptor ideation and route comparison were followed by a human-locked, deterministic fit.

S5.4 Verified and unresolved scope of PySR

The project archive contains historical PySR scripts and equation text files from an earlier phase, plus a later preregistration for a same-toolbox comparison. The later preregistration specifies, but by itself does not prove execution of, the configuration in table S7. No complete machine-readable result bundle matching the preregistered destination was located during this audit. Therefore no PySR accuracy, winning equation, run-time, failure count, or selection-frequency claim is reported here.

Table S7: PySR scope that is verifiable from `PYSR_PREREG.md`. These are preregistered settings, not a claim that every run completed.

Item	Preregistered scope
Input arms	Raw 127-channel arm; assisted 20-feature arm
Target	Decay-rate coordinate k
Search iterations	200
Populations and size	30 populations of 50
Maximum expression size	25
Binary operators	$+, -, \times, \div$
Unary operators	exp, log, square, and square root
Model selection	PySR `model_selection='best'`
Time cap	1,800 s per search
Planned random states	In-distribution: 0, 1, 2 for each arm; held-family search: 0
Planned search count	$2 \times 3 + 2 \times 5 = 16$ searches
Verified execution result	Unresolved; expected result bundle was not located in the audited artifacts
Use in final OLS relations	None; PySR was advisory and did not supply the reported terms or coefficients

The older phase-3 outputs predate the frozen bench protocol and cannot be silently relabelled as results of this preregistered comparison. Any future manuscript statement about exact PySR hyperparameters or performance should cite the archived completed run rather than this preregistration alone.

S6 Protocol-locked performance, uncertainty, and integrity checks

S6.1 Residual porosity and simple baselines

The transformation to k and k_E normalizes attenuation by porosity; it does not mathematically remove every residual dependence on ϕ. On TRAIN, the squared Pearson correlation between rate and porosity was 0.002 for k and 0.298 for k_E. We therefore fitted simple baselines on TRAIN only and scored them on the unchanged

reporting VAL set (table S8). Family models used the six registered family labels, with one level absorbed by the intercept.

Table S8: TRAIN-fitted simple baselines evaluated on reused reporting VAL. Predictor counts exclude the intercept. The final relation is included for scale, not as a claim that the nested models are inferentially interchangeable.

Model	Predictors	Strength $R^2(k)$	Modulus $R^2(k_E)$
Porosity, linear	1	−0.003	0.232
Porosity, quadratic	2	−0.004	0.231
Family only	5	0.265	0.257
Family + common porosity slope	6	0.270	0.516
Family-specific porosity slopes	11	0.271	0.536
Final six-descriptor relation	6	0.606	0.822

Using $(R^2_{\text{relation}} - R^2_{\text{baseline}})/(1 - R^2_{\text{baseline}})$ as a descriptive incremental score, the final relations add 0.607/0.768 beyond the linear-porosity baseline and 0.460/0.616 beyond the family-specific-porosity baseline for strength/modulus. These contrasts support topology-sensitive information beyond the simple baselines while retaining the more accurate term "porosity-normalized." A threshold sensitivity, without refitting, gave the following scores:

Table S9: Reporting-VAL sensitivity after excluding observations below a minimum realized porosity. The TRAIN-fitted final relations were not refitted.

Minimum ϕ	Rows	Strength $R^2(k)$	Modulus $R^2(k_E)$
0.00	1,000	0.606	0.822
0.04	797	0.615	0.824
0.06	591	0.644	0.807
0.08	484	0.674	0.791
0.10	376	0.681	0.757

S6.2 Dependence-aware performance estimates

The final models fitted on all 8,040 TRAIN rows were evaluated on the 1,000 reused reporting-VAL rows. Dependence-aware intervals and grouped cross-validation are the primary internal robustness summaries; row-wise estimates are retained as secondary provenance (table S10). Fold dispersions are population standard deviations across five folds, not confidence intervals.

Table S10: Protocol-locked reporting performance and retrospective robustness audits. "Nested grouped" repeats descriptor selection inside each outer fold on the original 7,000 TRAIN rows; "fixed grouped" evaluates the published specifications on 8,040 TRAIN rows.

Quantity	Strength k	Modulus k_E
Reporting-VAL R^2	0.606	0.822
95% family-stratified seed-cluster interval	[0.495, 0.680]	[0.787, 0.849]
95% row-bootstrap interval (secondary)	[0.517, 0.686]	[0.797, 0.842]
Nested grouped selection CV, mean ± SD	0.549 ± 0.017	0.773 ± 0.024
Fixed-specification grouped CV, mean ± SD	0.562 ± 0.054	0.785 ± 0.014
Historical fixed-specification row CV, mean ± SD	0.574 ± 0.013	0.786 ± 0.011
Property-retention R^2	0.975	0.992
Maximum VIF	1.89	4.19

Rate-scale R^2 is the headline quantity. The property-retention values include the dominant porosity term and are complementary context rather than evidence that topology is almost completely predicted. The nested grouped estimates are retrospective internal sensitivities; reused VAL and the absence of an external cohort prevent an independent-confirmation claim.

S6.3 Audited post-freeze numerical results

The prospective audit passed after all computations were complete. The primary manifest contains 903 formal cases (three intact baselines and 900 porous predictions), and the rate manifest contains 146 formal new cases (two baselines and 144 porous predictions). All 1,049 formal cases have atomic completion records and matching hashes; the audit found zero missing, corrupt, unexpected or still-running case directories and no quality-control failure. Eight smoke cases passed the production-shaped gate but remain excluded from scientific analysis. Every prediction row carries the frozen specification identity `43a91b8fa19c4cdfdaedc38e5d990753f2eed480949306135fc7d6fa34654e0b`, and the evaluator contains no code path for reselection, refitting, form changes, threshold tuning or retired-TEST use.

Table S11: Audited intact baselines for the three prospective primary packings. The geometric interparticle void fraction ϕ_{ip} describes the intact particle discretization and is not added to the imposed deletion-field porosity ϕ.

Packing (seed)	Spheres	ϕ_{ip}	UCS (MPa)	Modulus (GPa)
P01 (104729)	14,802	0.497671	87.4686	38.8383
P02 (130363)	14,224	0.519868	66.7677	32.2944
P03 (169087)	14,702	0.501492	75.5663	36.7176

Table S12: Primary post-freeze performance across 900 porous cases. Bias is predicted minus measured. The interval is the 2,000-replicate hierarchical percentile interval obtained by resampling packing and then family–seed clusters. Only three packing identities are available, so packing-level uncertainty remains imprecise.

Outcome	R^2	95% interval	MAE	RMSE	Bias
Strength-decay rate k	0.516	[0.388, 0.603]	0.984	1.323	−0.284
Modulus-decay rate k_E	0.735	[0.657, 0.797]	0.328	0.471	−0.109
Strength decay $-\ln(\sigma_c/\sigma_{c0})$	0.952	[0.940, 0.963]	0.084	0.121	−0.029
Modulus decay $-\ln(E/E_0)$	0.973	[0.961, 0.983]	0.034	0.064	−0.014

Table S13: Primary decay-rate performance by prospective packing ($n = 300$ porous cases per packing). Bias is predicted minus measured. Rows are descriptive strata; no packing-specific coefficient was fitted.

	Strength-decay rate k				Modulus-decay rate k_E			
Packing	R^2	MAE	RMSE	Bias	R^2	MAE	RMSE	Bias
P01	0.508	0.929	1.265	−0.516	0.782	0.294	0.421	−0.097
P02	0.458	1.062	1.407	−0.572	0.678	0.361	0.525	−0.158
P03	0.526	0.960	1.291	+0.236	0.729	0.328	0.459	−0.072

The pooled primary result is therefore not a claim of uniform within-family prediction. Strength-rate performance is negative for Boolean and Mixed and low for the other four families; modulus-rate performance is positive for every primary family but remains weak for DFN and shows appreciable bias for Clustered and Network. Residual association with realized porosity is also family dependent (for example, the modulus-rate residual correlation is +0.699 for Boolean and −0.458 for DFN). These adverse strata are part of the reported scope boundary rather than exclusions.

The rate analysis combines 144 newly simulated porous rows at factors 0.5 and 2.0 with 72 preregistered factor-1 counterparts from the primary P01 campaign, giving 216 evaluation rows. This reuse is identity-matched and does not increase the number of new simulations. The frozen geometry-only predictions are identical for a given geometry across factors by construction.

Table S14: Primary decay-rate performance by defect family ($n = 150$ porous cases per family). Negative R^2 values are retained and denote performance worse than predicting the mean within the stated family. These global frozen predictions were not refitted within family.

	Strength-decay rate k				Modulus-decay rate k_E			
Family	R^2	MAE	RMSE	Bias	R^2	MAE	RMSE	Bias
Boolean	−0.174	0.490	0.646	+0.025	0.494	0.206	0.257	−0.048
Chain	0.149	0.897	1.225	−0.038	0.800	0.194	0.238	−0.016
Network	0.082	1.027	1.292	−0.072	0.549	0.406	0.529	−0.219
DFN	0.301	1.113	1.379	−0.766	0.221	0.404	0.538	+0.047
Clustered	0.248	1.260	1.640	−0.524	0.633	0.461	0.706	−0.304
Mixed	−0.113	1.117	1.523	−0.328	0.728	0.294	0.373	−0.113

Table S15: P01 rate-arm decay-rate performance by strain-rate factor and in the pooled 216-row evaluation. Factor 1 rows are preregistered counterparts from the primary arm. Bias is predicted minus measured. Factor-specific scores are descriptive; no rate-arm uncertainty interval or between-factor hypothesis test is claimed.

		Strength-decay rate k				Modulus-decay rate k_E			
Factor	n	R^2	MAE	RMSE	Bias	R^2	MAE	RMSE	Bias
0.5	72	0.564	0.796	1.105	−0.049	0.818	0.270	0.363	−0.048
1.0	72	0.581	0.737	1.045	−0.128	0.819	0.272	0.362	−0.045
2.0	72	0.564	0.803	1.105	−0.292	0.818	0.272	0.364	−0.044
Pooled	216	0.571	0.779	1.085	−0.156	0.819	0.271	0.363	−0.046

Table S16: Pooled rate-arm decay-rate performance by family ($n = 36$ evaluation rows per family). Negative R^2 values are retained and denote performance worse than predicting the mean within the stated family; the pooled score must not be interpreted as family-uniform rate robustness.

	Strength-decay rate k				Modulus-decay rate k_E			
Family	R^2	MAE	RMSE	Bias	R^2	MAE	RMSE	Bias
Boolean	−0.278	0.417	0.515	+0.050	0.244	0.227	0.268	−0.061
Chain	−1.403	0.472	0.614	−0.049	0.775	0.197	0.273	+0.050
Network	−0.929	0.949	1.367	+0.651	−0.633	0.332	0.481	+0.060
DFN	0.663	0.885	1.053	−0.300	0.577	0.404	0.496	−0.035
Clustered	0.388	0.715	1.046	−0.118	0.910	0.201	0.275	−0.092
Mixed	−1.041	1.235	1.534	−1.172	0.654	0.267	0.305	−0.198

Table S17: Paired measured rate sensitivity relative to the nominal factor-1 P01 response ($n = 72$ structures per comparison). Relative change is computed only against nonzero references. Predictions are unchanged across factors because strain rate is not an input.

	Strength-decay rate k			Modulus-decay rate k_E		
Factor	Mean signed	Mean absolute	Mean relative	Mean signed	Mean absolute	Mean relative
0.5	−0.0792	0.2344	−0.95%	+0.0029	0.0084	+0.06%
2.0	+0.1633	0.2629	+1.92%	−0.0007	0.0083	−0.02%

The measured strength-rate shifts are modest on average but have a wider upper tail than those for modulus. The 95th percentiles of absolute k change were 0.812 at 0.5 times and 0.922 at 2.0 times; the corresponding k_E values were 0.032 and 0.034. Because the predictor cannot respond to strain rate, this arm quantifies outcome sensitivity and fixed-prediction error within a narrow P01 perturbation; it does not demonstrate rate-aware adaptation or invariance in other DEM or physical systems. The negative rate-family scores, particularly Network for both targets, preclude a claim of uniform family robustness.

S6.4 Bootstrap and permutation procedures

The headline reporting-VAL interval used $B = 2{,}000$ family-stratified cluster resamples. Family–seed clusters were resampled with replacement within each family, all 20 porosity rows of each selected cluster were retained, and the fitted TRAIN model was held fixed. The resulting percentile intervals were $[0.495, 0.680]$ for strength and $[0.787, 0.849]$ for modulus. They preserve the observed family counts and address within-seed dependence, but they do not include descriptor reselection, coefficient refitting, or uncertainty from the single intact packing.

For comparison with archived reporting, a nonparametric row bootstrap with $B = 2{,}000$ [3] sampled the 1,000 measured-predicted VAL pairs with replacement and gave $[0.517, 0.686]$ and $[0.797, 0.842]$. These narrower row-level intervals are secondary because they ignore family–seed dependence.

Standardized coefficient intervals used a different $B = 2{,}000$ procedure. Each descriptor was standardized once with its mean and population standard deviation over all 8,040 TRAIN rows, while the target remained unscaled. OLS with an intercept was then refitted on TRAIN-row bootstrap samples drawn with replacement using random seed 20260628; descriptors were not restandardized within each resample. The 2.5th and 97.5th percentiles are reported in table S18. They describe standardized descriptor effects and must not be conflated with the fixed-model VAL performance intervals. Intercept uncertainty was not estimated in this artifact.

Table S18: Standardized descriptor effects and 95% TRAIN-row bootstrap intervals. Raw coefficients are included only to map the effects to the published equations.

Target	Descriptor	Standardized effect [95% interval]	Raw coefficient
Strength	k_{mincut}	0.714 [0.659, 0.767]	0.5008
Strength	`dg_Amax`	0.245 [0.207, 0.283]	2.1087
Strength	`branch_den`	0.513 [0.470, 0.556]	2.9905
Strength	`dg_aucC`	0.443 [0.391, 0.495]	0.7049
Strength	`loc_normal_z`	0.204 [0.171, 0.240]	4.3107
Strength	`dg_xiclust`	0.195 [0.171, 0.219]	0.8889
Modulus	k_{mincut}	0.278 [0.261, 0.296]	0.1953
Modulus	`branch_den`	0.516 [0.491, 0.540]	3.0097
Modulus	`dg_AL`	0.178 [0.167, 0.190]	5.7505
Modulus	`bnd_coord_mean`	−0.175 [−0.191, −0.160]	−0.6411
Modulus	`skel_frac`	−0.204 [−0.220, −0.187]	−9.6168
Modulus	`dg_S2`	0.073 [0.061, 0.085]	1.9412

S6.5 Nested selection stability

The nested grouped audit also records how often each published descriptor reappeared across the five outer-fold selections (table S19). The complete published set recurred in three of five folds for each target when the archived equality `coord_num`=`bnd_coord_mean` is treated as an alias. The minimum-cut term appears in every fold by design because it was pinned. Pore-skeleton branch density, in contrast, was not pinned and recurred in four of five folds for both targets. Thus $\{k_{\mathrm{mincut}}, \rho_{\mathrm{br}}\}$ is described as a shared fitted motif, not a wholly independent discovery or a unique causal kernel.

The fixed-specification permutation control used 500 repetitions. In each repetition, the TRAIN target was permuted across rows, the already frozen six-descriptor OLS specification was refitted, and R^2 was evaluated on unchanged reporting-VAL rows. The 95th null percentiles were 0.0180 for strength and 0.0067 for modulus. None of the 500 scores reached the observed score. Because descriptor selection was not repeated, this is a frozen-specification null rather than a correction for the complete preceding search. We do not report $p = 0$; the plus-one Monte Carlo value is $p = (0 + 1)/(500 + 1) = 1/501 \approx 0.002$.

Table S19: Published-term recurrence in five outer folds of the retrospective nested family–seed grouped analysis. Frequencies count selected outer-fold specifications; k_{mincut} was pinned.

Strength descriptor	Folds	Modulus descriptor	Folds
k_{mincut} (pinned)	5/5	k_{mincut} (pinned)	5/5
`dg_Amax`	5/5	`dg_AL`	5/5
`dg_xiclust`	5/5	`skel_frac`	5/5
`loc_normal_z`	5/5	`branch_den`	4/5
`branch_den`	4/5	`coord_num/bnd_coord_mean`	4/5
`dg_aucC`	3/5	`dg_S2`	3/5

S6.6 Minimum-cut pinning sensitivity

The human decision to pin k_{mincut} was tested with the same original 7,000-TRAIN, six-descriptor, nested 5×5 family–seed-grouped selection protocol. "Eligible" made k_{mincut} optional and swappable; "excluded" removed it from the pool. No VAL or TEST outcome entered selection. The optional search selected k_{mincut} in all five outer folds and the full-TRAIN solution for both targets (table S20). The pinned and eligible strength paths were identical. For modulus, feature ordering changed the greedy/swap trajectory even though the final full-TRAIN set was the same up to order. Exclusion left grouped strength performance nearly unchanged but materially reduced modulus performance; its lower reused-VAL scores are secondary because VAL is reused.

Table S20: Sensitivity to the human minimum-cut prior. Nested and full-selection grouped scores use original TRAIN only; reporting VAL is reused and not independent. SD is the population standard deviation over five outer folds.

Target	Mode	Nested R^2	Pooled OOF R^2	Full grouped R^2	VAL R^2	k_{mincut} outcome
Strength	Pinned	0.549 ± 0.017	0.551	0.561	0.603	forced
Strength	Eligible	0.549 ± 0.017	0.551	0.561	0.603	5/5 folds + full
Strength	Excluded	0.550 ± 0.019	0.550	0.559	0.544	unavailable
Modulus	Pinned	0.773 ± 0.024	0.773	0.778	0.819	forced
Modulus	Eligible	0.759 ± 0.015	0.759	0.778	0.819	5/5 folds + full
Modulus	Excluded	0.723 ± 0.022	0.725	0.730	0.713	unavailable

S6.7 Retained-descriptor association and conditional effects

Table S21 deliberately separates four questions. Spearman ρ is a marginal association; the standardized coefficient is conditional on the other five retained terms; VIF diagnoses linear dependence; and remove-one loss is the decrease in mean five-fold family–seed-grouped TRAIN R^2 after deleting one term from the otherwise frozen specification. Descriptor selection was not repeated after deletion. None of these quantities is a causal, universal or optimal-feature-ranking measure.

Table S21: TRAIN-only interpretation statistics for the retained descriptors. "CV loss" is the decrease from the frozen six-term grouped-CV mean.

Target	Descriptor	Spearman ρ	Standardized coefficient	VIF	Grouped-CV loss
Strength	k_{mincut}	0.701	0.714	1.82	0.070
Strength	$A_{\max}$	0.532	0.245	1.67	0.008
Strength	ρ_{br}	0.467	0.513	1.67	0.044
Strength	AUC[C]	0.470	0.443	1.89	0.028
Strength	$\langle\lvert n_z\rvert\rangle_{\text{loc}}$	0.108	0.204	1.08	0.010
Strength	ξ_{clust}	0.449	0.195	1.26	0.008
Modulus	k_{mincut}	0.639	0.278	1.83	0.064
Modulus	ρ_{br}	0.777	0.516	4.19	0.093
Modulus	A_L	0.090	0.178	1.21	0.038
Modulus	$\bar{Z}_{\text{bnd}}$	−0.545	−0.175	2.69	0.017
Modulus	f_{skel}	0.354	−0.204	1.76	0.034
Modulus	S_{nem}	0.381	0.073	1.42	0.005

The sign reversal of f_{skel} from positive marginal association to negative conditional coefficient is a suppression pattern within a correlated modulus block. It does not imply that increasing pore-skeleton fraction directly increases the specimen modulus or causes lower attenuation. Conversely, the negative marginal and conditional signs for $\bar{Z}_{\text{bnd}}$ are consistent with more surviving initial contacts supporting elastic transfer, but remain observational within the generated corpus.

S6.8 Functional-form and shared-scaling sensitivities

The fixed reported descriptor sets were refitted under three retention forms: $r = \exp[-\phi a(\mathbf{g})]$, $r = (1-\phi)^{a(\mathbf{g})}$, and $r = \exp[-\phi^p a(\mathbf{g})]$. In the stretched form, $p \in \{0.5, 0.6, \ldots, 1.5\}$ was selected solely within nested grouped TRAIN folds by retention RMSE. Coefficients were fitted by OLS on the corresponding transformed target, and predictions were evaluated without clipping on the original retention scale. The modest differences in table S22 do not identify a unique mechanistic porosity form.

Table S22: Alternative porosity forms with fixed descriptors. OOF values are pooled family–seed-grouped TRAIN predictions; VAL is reused.

Target	Form	Selected p	OOF retention R^2	OOF RMSE	VAL retention R^2
Strength	Exponential	—	0.96667	0.03941	0.97489
Strength	Power	—	0.96594	0.03984	0.97432
Strength	Stretched exponential	1.2	0.96741	0.03897	0.97486
Modulus	Exponential	—	0.99022	0.01891	0.99238
Modulus	Power	—	0.99013	0.01900	0.99242
Modulus	Stretched exponential	1.0	0.99022	0.01891	0.99238

To assess whether this pooled result masked family-dependent form preferences, we repeated the comparison separately within each family on the original observation-level retention scale. The analysis was restricted to TRAIN: the globally fixed descriptor identities were retained, coefficients were refitted within family, and outer folds held out complete family–seed groups. For the stretched exponential, p was selected in nested inner grouped folds; the separately reported full-family TRAIN value of p did not enter the out-of-fold predictions. Reporting VAL and retired TEST were excluded. Accordingly, table S23 is a conditional sensitivity of attenuation form and within-family coefficient refitting, not an independent validation or evidence that any listed form is a unique physical mechanism.

Table S23: Family-specific attenuation-form sensitivity on the original retention scale. The six descriptor identities were held fixed globally, while coefficients were refitted within each family. Each outer fold held out complete family–seed groups. The stretched exponent was selected only in inner within-family TRAIN folds; the displayed p is the separate full-family TRAIN selection and was not used to construct the OOF score. Reporting VAL and retired TEST are excluded. These are conditional form sensitivities, not independent family validations.

Target	Family	Rows	Groups	Grouped OOF retention R^2			Full-TRAIN p
				Exponential	Power	Stretched exponential	
Strength	Boolean	1560	78	0.99213	0.99215	0.99214	1.1
Strength	Chain	1560	78	0.97587	0.97519	0.97577	0.8
Strength	Clustered	1560	78	0.95075	0.95025	0.95490	1.4
Strength	DFN	300	15	0.93495	0.93476	0.93195	1.0
Strength	Mixed	1500	75	0.97447	0.97404	0.97536	1.3
Strength	Network	1560	78	0.96042	0.95966	0.96313	1.4
Modulus	Boolean	1560	78	0.99800	0.99797	0.99808	0.9
Modulus	Chain	1560	78	0.99618	0.99615	0.99614	1.0
Modulus	Clustered	1560	78	0.98348	0.98266	0.98306	0.8
Modulus	DFN	300	15	0.98250	0.98155	0.98711	0.5
Modulus	Mixed	1500	75	0.99329	0.99331	0.99329	1.0
Modulus	Network	1560	78	0.98935	0.98950	0.98955	1.1

Because both the response rates and k_{mincut} divide a logarithmic attenuation by ϕ, we separately tested their shared scaling. The Pearson correlation between k_{mincut} and the response rate was 0.673 for strength and 0.621 for modulus; after linear residualization against ϕ and family indicators it remained 0.476 and 0.608. Fixed-specification grouped TRAIN results in table S24 show that unnormalized cut attenuation or retention did not reproduce the complete result and often violated the registered VIF threshold. The normalized channel is therefore statistically better conditioned, while the remaining association cannot be attributed solely to the common denominator.

Table S24: Shared-scaling sensitivity on 8,040 TRAIN cases. Values are mean ± population SD over five family–seed-grouped folds; feature sets are fixed and selection is not repeated.

Cut-channel variant	Strength R^2	Modulus R^2	Maximum VIF (strength/modulus)
Published normalized cut rate	0.562 ± 0.054	0.785 ± 0.014	1.89 / 4.19
Raw cut attenuation	0.511 ± 0.044	0.777 ± 0.021	4.40 / 7.16
Raw cut retention	0.517 ± 0.046	0.762 ± 0.019	6.24 / 11.55
Cut channel excluded	0.491 ± 0.040	0.721 ± 0.025	1.64 / 3.05

S6.9 Four nonpositive Δk observations

The original 7,000 TRAIN rows contain 6,996 cases with $\Delta k = k - k_E > 0$ and four with $\Delta k \leq 0$ (table S25). All four lie at the two lowest registered porosity levels; three are Chain cases, and Chain seed 36 recurs at adjacent levels. They remain in every analysis. Small ϕ magnifies modest raw retention differences in the rate contrast, but this fact alone cannot classify the observations as numerical noise. Their existence rules out a universal positive-sign constraint and the interpretation of Δk as a measured damage variable.

Table S25: Complete TRAIN-only ledger of nonpositive Δk observations. $\Delta r = (\sigma_c/\sigma_{c0}) - (E/E_0)$.

Case	Family	ϕ	k	k_E	Δk	Δr
`phase2_Boolean_phi020_seed5`	Boolean	0.020094	3.2596	3.9935	−0.7339	0.01371
`phase2_Chain_phi020_seed10`	Chain	0.020039	3.7780	3.8649	−0.0869	0.00161
`phase2_Chain_phi020_seed36`	Chain	0.020086	2.6417	3.4759	−0.8343	0.01576
`phase2_Chain_phi025_seed36`	Chain	0.025016	2.8333	3.4076	−0.5744	0.01329

S6.10 VIF and deterministic integrity checks

VIF was computed for the final six-descriptor design of each empirical relation. Maximum values were 1.89 for strength and 4.19 for modulus, satisfying the locked threshold below 5. The modulus block is nevertheless sufficiently correlated that individual coefficient signs, especially the negative f_{skel} term, are interpreted conditionally.

The deterministic gate checked partition counts, absence of missing selected descriptors in TRAIN and VAL, split hashes, feature/label separation, exact reproduction of the matched 9,000-case fit before the top-up, equality of `coord_num` and `bnd_coord_mean`, coefficient signs, and VIF. A language model could advise on report completeness, but only deterministic checks could trigger PASS. The exact public split hashes should be copied from the release manifest when a public accession is assigned; no hash is invented in this document.

S7 Matched baselines and implementation settings

All comparator values use the original matched 9,000-case frame: 7,000 TRAIN, the same 1,000 reporting VAL, and an excluded 1,000-case retired TEST partition. The baseline frame is not mixed with the 10,040-case headline refit.

Table S26 records the reporting-VAL scores used in Figure 4a and additionally reports gains over the matched Minkowski-only score for the same target; those gains are arithmetic differences on one reporting scale. Figure 4c instead plots the absolute development-minus-reporting-VAL magnitudes: strength/modulus values are 0.053/0.057 for Minkowski-only, 0.038/0.035 for the six-descriptor relations, 0.052/0.048 for the remediated CNN, and 0.147/0.086 for GBM. Minkowski-only, the relations and GBM use mean row-wise five-fold TRAIN cross-validation as the development estimate, whereas CNN uses the winning configuration's grouped internal-TRAIN holdout score. Absolute values discard direction, and the development estimators remain heterogeneous; the panel is a provenance diagnostic rather than a common stability, generalization or overfitting metric.

On this matched frame, the six-descriptor relations attained $R^2(k) = 0.603$ with fixed-model, 2,000-resample VAL-row bootstrap interval $[0.515, 0.684]$ and five-fold TRAIN $R^2 = 0.565$, and $R^2(k_E) = 0.819$ with interval $[0.794, 0.840]$ and five-fold TRAIN $R^2 = 0.784$. These are distinct from the final 8,040-TRAIN refit values reported in the main headline table. All geometry-based representations originate from the same reconstructed specimen; six is the final predictor dimension after descriptor computation, not the cost of generating those descriptors.

Table S26: Matched-frame reused-reporting-VAL results and gains over the Minkowski-only reference. The same four models and order are used in every Figure 4 panel. Input dimension is representation size after preprocessing, not imaging or descriptor-computation cost.

		Strength decay rate k		Modulus decay rate k_E	
Model	Input dimension	VAL	Gain	VAL	Gain
Minkowski only	4	0.167	0.000	0.382	0.000
Six-descriptor relation	6	0.603	0.436	0.819	0.437
3D CNN ensemble	128,000	0.428	0.261	0.686	0.304
GBM geometry+mechanics	122	0.656	0.489	0.823	0.441

S7.1 Minkowski-only OLS

The Minkowski-only ordinary-least-squares model used four inputs: Euler characteristic, Euler-characteristic density, surface density, and specific surface. Porosity itself was omitted because the target was already a porosity-normalized rate. Reporting-VAL R^2 was 0.167 for strength and 0.382 for modulus; corresponding row-wise five-fold TRAIN values were 0.220 and 0.439.

S7.2 Three-dimensional convolutional neural network

The target-specific 3D CNN was implemented in PyTorch [4]. It received the deterministically regenerated $40 \times 40 \times 80$ Boolean pore field, or 128,000 voxel inputs. The original three-seed result was superseded for revision by the fail-closed protocol in table S27: retired TEST was removed before voxel caching; a single 53-group internal holdout from the 7,000 TRAIN rows ranked a bounded four-configuration grid; the selected configuration and median-best epoch were frozen; five initialization seeds were then refitted on all TRAIN rows; and only then was reporting VAL opened once. The five prediction vectors were averaged for the ensemble score in Figure 4.

Table S27: Reviewer-remediated 3D-CNN protocol. Each target was selected and trained separately; all selection used TRAIN only.

Item	Configuration
Candidate grid	Four registered combinations of channel widths, learning rate, weight decay and dropout; one tuning seed per candidate
Grouped tuning split	5,940 fit rows (297 family–seed groups) and 1,060 holdout rows (53 groups); zero group overlap; seed 20260722
Convolutional body	Four $3 \times 3 \times 3$ convolutions, stride 2, padding 1; selected channels 32/64/128/256 for strength and 16/32/64/128 for modulus
Block operations	Convolution, batch normalization, ReLU
Head	Adaptive global average pooling; 64-unit linear layer; ReLU; selected dropout 0.3 (strength) or 0.1 (modulus); scalar output
Trainable weights	1,179,553 (strength); 299,537 (modulus)
Loss and optimizer	Mean squared error; AdamW
Selected learning rate	10^{-3} for both targets
Selected weight decay	10^{-5} (strength); 10^{-4} (modulus)
Batch size	32
Tuning limit	100 epochs; patience 15; minimum R^2 improvement 10^{-4}
Frozen all-TRAIN epochs	13 (strength); 8 (modulus)
Final initialization seeds	20000, 20001, 20002, 20003, 20004
Individual strength R^2	0.3637, 0.3805, 0.3591, 0.3641, 0.3901; mean 0.3715 ± 0.0118
Individual modulus R^2	0.6569, 0.7114, 0.6461, 0.6595, 0.5696; mean 0.6487 ± 0.0456
Five-network ensemble R^2	0.4278 (strength); 0.6856 (modulus)
Peak CUDA memory	382.1 MiB (strength); 230.4 MiB (modulus)

The selected grouped-TRAIN holdout scores were 0.4793 for strength and 0.7336 for modulus. Relative to the five-network ensemble reporting scores, the absolute differences were 0.0516 and 0.0481. They compare a candidate-selection run with an ensemble and therefore remain provenance diagnostics. A paired 5,000-replicate family-stratified seed-cluster bootstrap instead compared the fixed matched relation and fixed ensemble on the same reused-VAL resamples: relation-minus-CNN differences were 0.1754 with interval $[0.0274, 0.3143]$ for strength and 0.1330 with interval $[0.0324, 0.2369]$ for modulus. Positive values favour the compact relation.

These intervals condition on both fitted predictors, do not include training or search uncertainty, and cannot make VAL independent.

S7.3 Geometry-plus-mechanics gradient boosting

The high-information reference used scikit-learn's `HistGradientBoostingRegressor` [5, 6] with 48 archived geometry inputs and 74 archived mechanics or diagnostic inputs, totaling 122 input dimensions. These are input dimensions, not 122 fitted coefficients. The audited settings were: squared-error loss, maximum depth 3, learning rate 0.05, maximum 1,000 iterations, L_2 regularization 2.0, early stopping enabled with 15% internal validation and 30-iteration patience, minimum 20 samples per leaf, maximum 31 leaf nodes, 255 bins, and random seed 0. Reporting-VAL R^2 was 0.655610 for strength and 0.823017 for modulus. Shuffled five-fold TRAIN means with `random_state=2026` were 0.803011 and 0.909217, giving descriptive gaps of +0.147401 and +0.086200.

This model is a mechanics-assisted reference, not a clean geometry-only baseline. One available mechanics channel, `k_sm_u_m4`, is nearly collinear with the FFT-derived modulus-rate proxy `k_E_fft` (TRAIN-only Pearson $r = 0.985$). This value does not describe correlation with the DEM modulus-rate target: the corresponding TRAIN-only correlation with k_E is $r = 0.759$. Consequently, the modulus GBM result is not evidence of clean static-geometry generalization. Approximate leaf counts mentioned in older captions are omitted because the exact fitted-model extraction was not present in the audited result bundle.

S8 Discussion-stage paired-rate and held-family diagnostics

S8.1 Interpretive scope of the paired-rate figure

Figure 5a is a conceptual working model, not a measured force-network or damage-field reconstruction. The two sketches refer to the same initial particle–bond geometry at different loading stages. They motivate the falsifiable possibility that initial topology largely sets the pre-peak load-sharing response, whereas progressive bond breaking and localization contribute preferentially to the difference between strength and modulus attenuation. The construction does not establish that causal sequence and remains compatible with missing static descriptors, contact-law effects, and estimator coupling.

The contrast $\Delta k = k - k_E$ in Fig. 5 is an exact algebraic decomposition of the two fitted specimen-level rates. It is not a third fitted relation and must not be called a damage rate. Figure 5b evaluates the exact normalized-retention expressions at the original 7,000-TRAIN medians, $k_E = 4.8269$ and $\Delta k = 3.1477$, solely to illustrate how the two rate components separate with increasing porosity; the shaded gap is not an uncertainty interval. Figure 5c plots all 7,000 original TRAIN observations in $(k_E, \Delta k)$ coordinates. Of these, 6,996 have $\Delta k > 0$. Large points are family centroids, dotted contours follow constant $k = k_E + \Delta k$, and ellipses are Gaussian-equivalent descriptive 50% covariance regions rather than confidence regions or exact empirical contours.

Figure 5d is a post-hoc coefficient-portability stress test. Descriptors and functional forms were fixed globally before coefficients were refitted on the pooled TRAIN+VAL cases from four pure families and scored on pooled TRAIN+VAL cases from the held pure family. Mixed and retired TEST were excluded. Because every family influenced the earlier global descriptor and form development, this calculation is not end-to-end leave-one-family-out discovery, OOD evaluation, zero-shot transfer, or independent validation.

S8.2 Final global relations by reporting family

Table S28 reports the final 8,040-TRAIN global relations by reporting family without family-specific refitting. Each pure-family block comprises five family–seed groups and Mixed comprises 25. Negative R^2 is retained rather than clipped. The unweighted mean across the five pure-family R^2 values is 0.067 for strength and 0.612 for modulus; consequently, the global strength score should not be read as uniform within-family accuracy. Bias is mean predicted minus measured rate.

Table S28: Family-resolved reporting-VAL performance of the final global relations. No family-specific refitting is performed.

Family	Rows	Seed groups	R^2	MAE	RMSE	Bias
Strength decay rate k						
Boolean	100	5	−0.272	0.461	0.576	+0.103
Chain	100	5	0.018	0.852	1.221	+0.016
Network	100	5	0.236	0.825	1.000	−0.038
DFN	100	5	0.370	1.149	1.559	−0.296
Clustered	100	5	−0.018	1.112	1.350	−0.676
Mixed	500	25	0.575	0.837	1.183	−0.108
Overall	1,000	50	0.606	0.858	1.186	−0.143
Modulus decay rate k_E						
Boolean	100	5	0.455	0.192	0.245	+0.025
Chain	100	5	0.646	0.250	0.309	+0.215
Network	100	5	0.672	0.309	0.395	−0.191
DFN	100	5	0.487	0.380	0.505	−0.071
Clustered	100	5	0.801	0.308	0.400	−0.175
Mixed	500	25	0.812	0.255	0.350	−0.050
Overall	1,000	50	0.822	0.272	0.366	−0.045

S8.3 Legacy equal-input within-family sensitivity

This legacy sensitivity used the original matched 7,000-TRAIN frame, before the later 1,040-case TRAIN-only top-up. For each pure family and target separately, all 48 legacy static-geometry descriptors were standardized using that family's TRAIN cases. Ridge regression selected its penalty by `RidgeCV` from 25 logarithmically spaced values between 10^{-3} and 10^{3}, then was scored only on that family's reused reporting-VAL cases. Boolean, Chain, Network, and Clustered each contained 1,300 TRAIN and 100 VAL rows; DFN contained 300 TRAIN and 100 VAL rows. Mixed and TEST were excluded. This legacy pool includes as-built coordination and predates the final multiscale descriptor system, so table S29 is a sensitivity analysis rather than another evaluation of the reported six-descriptor relations.

Table S29: Legacy 48-descriptor within-family ridge results on reused reporting VAL. Means are unweighted across the five pure families.

Family	Strength $R^2(k)$	Modulus $R^2(k_E)$
Boolean	0.2023	0.8497
Chain	0.4565	0.7521
Network	0.1148	0.6982
DFN	0.0785	0.3644
Clustered	0.5022	0.7514
Unweighted mean	0.2709	0.6832

S8.4 Compact versus full tested static geometry

The compact-to-full static-geometry comparison used the same matched 7,000 TRAIN and 1,000 reused reporting-VAL rows. The full tested pool contained 60 static geometric descriptors, including the multiscale `dg_` fields; historical `coord_num` was removed because it exactly duplicates `bnd_coord_mean`. Features were standardized with TRAIN means and standard deviations, and the ridge penalty was selected from 40 logarithmically spaced values between 10^{-3} and 10^{4}. Strength $R^2(k)$ increased from 0.603 for the six-descriptor OLS relation to 0.697 for the 60-descriptor ridge model; modulus $R^2(k_E)$ increased from 0.819 to 0.846. The displayed differences, +0.094 and +0.027, use the archived three-decimal endpoints. They are descriptive reused-VAL comparisons and do not define a physical ceiling.

S8.5 DFN sampling caveat

DFN received neither TRAIN-only top-up. Its final ledger contains 300 TRAIN, 100 reporting VAL, and 100 retired TEST cases, compared with 1,560 TRAIN cases for each topped-up pure family. In descriptive TRAIN+VAL analyses the DFN pool therefore contains 400 rows; no synthetic top-up or balancing weight was introduced. Mixed has no role in the five-pure-family coefficient-refit diagnostic.

S8.6 What the held-family calculation does

The post-hoc diagnostic in the discussion figure is not end-to-end leave-one-domain-out discovery. The six descriptors and functional form had already been selected with project-wide access to all families. For each held pure family, only coefficients were refitted on the pooled TRAIN+VAL rows of the other four pure families, and the held family's pooled TRAIN+VAL rows were scored. Mixed and retired TEST were excluded.

Table S30: Post-hoc held-family coefficient-refit stress test with globally fixed descriptors and functional forms. Negative R^2 is retained and denotes performance worse than predicting the held-family mean; the calculation is not an unseen-family validation.

Held family	Strength $R^2(k)$	Modulus $R^2(k_E)$
Boolean	−4.240	+0.491
Chain	−0.353	+0.620
Network	+0.162	+0.668
DFN	+0.181	+0.198
Clustered	+0.280	+0.675

Positive point estimates occur in three of five strength held-family evaluations and all five modulus evaluations. These values support only a discussion-stage statement that coefficient refitting is more stable for modulus than strength within the tested family system. They are not OOD validation, zero-shot transfer, new-family prediction, family-level inference, or external generalization. The DFN value is especially sensitive to its smaller pool. No mechanism is proven by this diagnostic.

S9 Experimental translation and observability roadmap

The present study contains no experimental validation. A defensible test would prospectively freeze segmentation, descriptor computation, coefficients and acceptance rules before acquiring a specimen-matched imaging–mechanics cohort. Each specimen would be imaged in three dimensions before loading, then tested for pre-peak modulus and uniaxial compressive strength under a registered protocol. Intermittent micro-CT, digital volume correlation or acoustic-emission localization could test the proposed distinction between initial load sharing and strength-specific failure evolution. Fracture toughness would require a separate notched-specimen protocol and must not be inferred from UCS or contact tensile strength.

Table S31: Experimental observability boundary for the reported descriptor families and paired-response hypothesis.

Quantity	Plausible measurement	Required qualification
Defect porosity, connected-component size and surface geometry	Segmented 3D X-ray CT or FIB–SEM volume	Resolution, threshold and partial-volume sensitivity must be preregistered.
Pore skeleton, branch density and multiscale correlations	Deterministic analysis of the segmented defect field	Skeletonization and scale conventions must match or be recalibrated; they are not instrument readings.
Orientation and local-normal descriptors	Segmented surface mesh or voxel field	Loading-axis registration and anisotropy convention must be fixed before outcome access.
Minimum-cut capacity	Reconstructed solid/contact graph or an explicitly defined continuum proxy	The DEM cohesive-contact graph is not directly visible in CT; an experimental proxy requires independent physical justification and cannot inherit the DEM coefficient automatically.
Boundary coordination and regenerated-bond quantities	Particle/contact reconstruction where grains are resolvable	Not directly observable in most continuum porous solids; omission or a preregistered proxy is required.
Modulus and UCS retention	Same-specimen mechanical tests with an intact reference population	Loading rate, specimen size, moisture and material batch must be controlled; uncertainty in the reference must propagate.
Failure evolution and Δk hypothesis	Time-lapse CT, digital volume correlation, acoustic emission or post-mortem crack mapping	These data test the proposed mechanism; Δk itself remains an algebraic response contrast, not a measured damage variable.
Fracture toughness	Independently calibrated notched-specimen test	Geometry and stress-intensity/energy convention must be specified; no value is supplied by the present JCFpm inputs.

S10 Submitted reproducibility archive

The submitted Supplementary Data Archive uses the following reader-facing layout. These are the paths present in the submitted ZIP rather than private workstation locations:

- the TEST-free 9,040-row analysis table, label-free 10,040-case split manifest, data dictionary and archived intact baseline are under `data/`;
- the frozen relation specification, portable verification code and selected analysis snapshots are under `code/analysis/`, with the resulting public verification record at `data/verification_report.json`;
- the grouped CNN protocol, runner, final model record and 1,000-row reporting-VAL prediction ledger are under `code/cnn/`;
- minimum-cut, descriptor, attenuation-form, Δk, CNN-completion and bibliography audits are under `results/`, with corresponding archival source snapshots under `code/reviewer_analyses_archival/`;
- the post-freeze DEM protocol, manifests and frozen evaluator are under `code/prospective_dem/`, while audited case summaries and prediction ledgers are under `data/prospective/` and the integrated verification record is `results/prospective_results_verified.json`;
- final figure assets, editable vectors where retained, plotting scripts and available source snapshots are under `figures/`;
- sanitized proposer–critic prompts, logs, manifests and checksums are under `provenance/`;
- recorded software versions and seeds are under `environment/`; and
- `SOURCE_MANIFEST.json`, `SHA256SUMS.tsv` and `code/public_release_self_test.py` provide inventory, integrity and portable verification gates.

The archive does not present the retained archival snapshots as end-to-end regeneration scripts when they depend on unavailable historical voxel arrays or retired-TEST outcomes. The portable self-test is the supported public verification route and preserves the stated data boundary.

The `Supplementary_Data_Archive/` directory inside `materials-4491511_supplementary_materials.zip` includes immutable split hashes; environment and dependency records; the processed feature/response ledger; generator manifests; final coefficients; reviewer-audit scripts and machine-readable outputs; prospective case manifests, completion/failure records and prediction tables; figure source data; and available LLM request

Table S32: Recorded computational environments. An unavailable historical version is reported as unavailable rather than inferred from a current installation.

Stage	Recorded software and version information
Original 10,040-case DEM corpus	YADE with JCFpm; the exact executable version string was not retained in immutable run metadata. The archived configuration, generator code, seeds, case ledger, and processed responses define the recoverable implementation boundary.
Prospective post-freeze DEM campaign	YADE `2026-05-27.git-814fcf6`; embedded Python 3.11.15. The preflight record and executable identity accompany the prospective archive.
Tabular fitting and reviewer-requested audits	Python 3.12.7; NumPy 1.26.4; pandas 2.2.2; SciPy 1.13.1; scikit-learn 1.5.1; scikit-image 0.24.0.
Reviewer-remediated 3D CNN	Python 3.12.7; NumPy 1.26.4; pandas 2.2.2; PyTorch 2.7.0+cu118; CUDA runtime 11.8; cuDNN 90100; NVIDIA GeForce RTX 4060 Laptop GPU.
Final statistical graphics	Matplotlib 3.9.2 in the analysis environment above; figure source data and plotting scripts are included in the revision archive where available.

metadata and prompt checksums. A future public release should preserve this documented scope and add repository and archival accession identifiers after assignment. A machine-readable completed PySR bundle is required only if a PySR performance number is discussed. Missing historical metadata remains explicitly identified rather than inferred from current software defaults.

S11 Compact claim-boundary checklist

- The final estimators are OLS models with intercepts, not symbolic-regression equations selected by an agent.
- Each published empirical relation contains six descriptors and seven fitted coefficients including the intercept.
- The 1,000-case VAL partition is a reused reporting benchmark; it is not fresh, external, or independent validation.
- The reported empirical relations were not evaluated on retired TEST. Project-wide TEST handling is not described as untouched because of the historical indirect reward path disclosed in section S5.3.
- The original selector used row-wise folds; the retrospective headline sensitivity repeats selection in nested family–seed grouped folds on the original 7,000 TRAIN rows.
- Headline performance intervals use 2,000 family-stratified seed-cluster resamples; archived row-bootstrap intervals and TRAIN-row coefficient refits are secondary diagnostics.
- The 500-permutation analysis tests the frozen specification and yielded zero exceedances; it is not reported as $p = 0$.
- The GBM has 122 inputs, not 122 fitted parameters, and its mechanics-assisted modulus result is near-target.
- The held-family calculation is a post-hoc coefficient-refit diagnostic, not OOD or zero-shot validation.
- The exponential porosity form is a parsimonious parameterization, not a uniquely established physical law.
- The minimum-cut channel is a human-admitted prior; optional and excluded replays quantify its influence.
- Positive Δk is a dominant pattern with four retained exceptions, not a universal sign constraint.
- The post-freeze primary campaign contains 900 porous predictions from three new packing realizations; it is prospective numerical evidence within the frozen YADE/JCFpm framework, not experimental or broad external validation.
- The P01 rate arm tests only 0.5- and 2.0-times perturbations; frozen geometry-only predictions are rate-invariant by construction and do not learn the rate effect.
- All evidence is synthetic; external experimental validation remains future work.

References

[1] Janek Kozicki and Frédéric-Victor Donzé. A new open-source software developed for numerical simulations using discrete modeling methods. *Computer Methods in Applied Mechanics and Engineering*, 197(49–50):4429–4443, 2008. doi: 10.1016/j.cma.2008.05.023.

[2] Luc Scholtès and Frédéric-Victor Donzé. A DEM model for soft and hard rocks: Role of grain interlocking on strength. *Journal of the Mechanics and Physics of Solids*, 61(2):352–369, 2013. doi: 10.1016/j.jmps.2012.10.005.

[3] Bradley Efron. Bootstrap methods: Another look at the jackknife. *The Annals of Statistics*, 7(1):1–26, 1979. doi: 10.1214/aos/1176344552.

[4] Adam Paszke, Sam Gross, Francisco Massa, Adam Lerer, James Bradbury, Gregory Chanan, Trevor Killeen, Zeming Lin, Natalia Gimelshein, Luca Antiga, Alban Desmaison, Andreas Köpf, Edward Yang, Zachary DeVito, Martin Raison, Alykhan Tejani, Sasank Chilamkurthy, Benoit Steiner, Lu Fang, Junjie Bai, and Soumith Chintala. PyTorch: An imperative style, high-performance deep learning library. In *Advances in Neural Information Processing Systems 32*, pages 8024–8035, 2019. URL https://proceedings.neurips.cc/paper/2019/hash/bdbca288fee7f92f2bfa9f7012727740-Abstract.html.

[5] Fabian Pedregosa, Gaël Varoquaux, Alexandre Gramfort, Vincent Michel, Bertrand Thirion, Olivier Grisel, Mathieu Blondel, Peter Prettenhofer, Ron Weiss, Vincent Dubourg, Jake Vanderplas, Alexandre Passos, David Cournapeau, Matthieu Brucher, Matthieu Perrot, and Édouard Duchesnay. Scikit-learn: Machine learning in Python. *Journal of Machine Learning Research*, 12: 2825–2830, 2011. URL https://jmlr.org/papers/v12/pedregosa11a.html.

[6] Jerome H. Friedman. Greedy function approximation: A gradient boosting machine. *The Annals of Statistics*, 29(5):1189–1232, 2001. doi: 10.1214/aos/1013203451.
